# Improving the reactivity of phenylacetylene macrocycles toward topochemical polymerization by side chains modification

**DOI:** 10.3762/bjoc.10.167

**Published:** 2014-07-15

**Authors:** Simon Rondeau-Gagné, Jules Roméo Néabo, Maxime Daigle, Katy Cantin, Jean-François Morin

**Affiliations:** 1 Département de Chimie and Centre de recherche sur les Matériaux Avancés (CERMA), Université Laval, 1045 avenue de la Médecine, G1V 0A6 Québec, Québec, Canada

**Keywords:** carbon nanomaterials, organogels, phenylacetylene macrocycles, polydiacetylenes, topochemical polymerization

## Abstract

The synthesis and self-assembly of two new phenylacetylene macrocycle (PAM) organogelators were performed. Polar 2-hydroxyethoxy side chains were incorporated in the inner part of the macrocycles to modify the assembly mode in the gel state. With this modification, it was possible to increase the reactivity of the macrocycles in the xerogel state to form polydiacetylenes (PDAs), leading to a significant enhancement of the polymerization yields. The organogels and the PDAs were characterized using Raman spectroscopy, X-ray diffraction (XRD) and scanning electron microscopy (SEM).

## Introduction

The self-assembly of molecular building blocks is an increasingly popular method for the preparation of new semiconducting materials. Rational design of building blocks and their assembly using non-covalent interactions can provide control over the size, shape and electronic properties of the resulting nanoarchitectures [1–3]. It is therefore not surprising that supramolecular interactions are regularly used to afford long-range molecular organization for organic and molecular electronics applications in which high-level of organization is required to reach good charge transport properties [4–6]. However, supramolecular assemblies often suffer from poor stability, meaning that variation of the storage and device operation conditions can perturb the molecular organization, leading to a decline of the materials efficiency overtime [7]. In this regard, the covalent immobilization of supramolecular assemblies using physical stimuli (heat or light) is an interesting way to obtain stable, organized materials [8–11]. For example, self-assembled butadiyne-containing molecules can be polymerized in a gel or crystalline state to yield polydiacetylenes (PDAs) following a 1,4-addition mechanism, thus fixing the initial molecular organization through covalent bonds formation [12–13]. In order for this polymerization to proceed, the molecules within the assembly must be oriented relative to each other following critical parameters, namely a distance of ≤4.9 Å and a tilt angle of 45° between the monomers [12]. Thus, careful selection of functional groups onto the molecule is needed to reach these requirements [14–20].

Recently, many research groups used this strategy to design several types of π-conjugated monomers capable of hydrogen bonding to create nanowires [21–23], nanoparticles [24–27], nanotubes [28–31] and two-dimensional layered materials [32] from organogels. The key to success was to obtain a good balance between solubility and gelation properties. Moreover, subtle changes in the chemical nature of the building blocks can have a dramatic impact on the self-assembly process. This was exemplified in the case of nanotubes obtained from self-assembled butadiyne-containing macrocycles, which stack on top of each other in columnar fashion to give supramolecular nanotubular structures [33–38]. Among other things, we have shown that inversion of the amide group configuration (acetanilide vs benzamide) at two different positions on phenylacetylene macrocycles (PAMs) leads to significant changes of the gelation properties and, consequently, on the critical parameters needed for polymerization through 1,4-addition reaction [38]. In fact, the acetanilide configuration provides macrocycles which can barely self-assemble in organic solvents while the benzamide configuration yields macrocycles with much greater gelation properties that allow the formation of PDA-based nanorods. Nonetheless, the polymerization of diyne units within PAMs to give covalent nanorods and nanotubes is very slow and gives low yields of polymerization, generally too low to be determined accurately [38–39]. In order to increase the efficiency of polymerization, the incorporation of external diyne chains on the PAMs core have been realized [31]. This design allowed us to increase the yield of polymerization to 15%. However, the addition of external diyne units is synthetically tedious and a more efficient strategy to significantly increase the yield of the topochemical polymerization is necessary.

**Figure 1:**
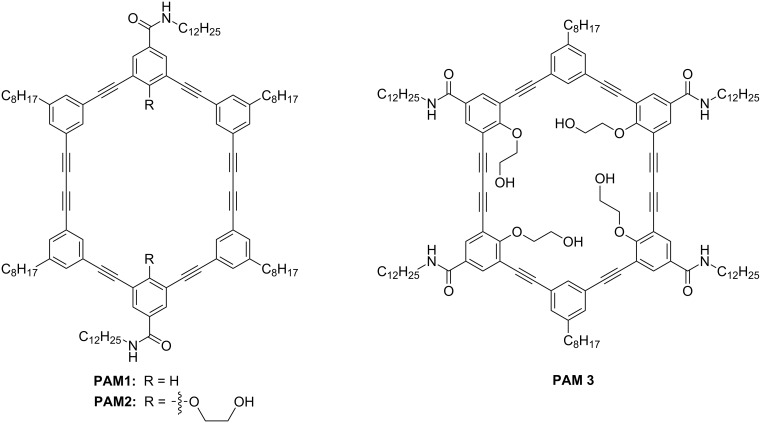
Structures of **PAM1** to **PAM3**.

In order to take a step further toward a better comprehension of the self-assembly of PAMs and to increase the polymerization yield, we decided to introduce substituents on the inside of the macrocycles, more precisely a polar 2-hydroxyethoxy group. Such a polar group proved to be useful to increase the intermolecular interactions between PAMs through hydrogen bonding in the solid state [40–41]. We hypothesized that this structural change could provide better control over the molecular organization of PAMs and, consequently, could lead to higher yield of PDA-walled nanorods through PAMs polymerization. Herein, we report the synthesis, gelation properties and topochemical polymerization of a new series of PAMs (**PAM2** and **PAM3**, Figure 1) with polar side chains pointing inside the macrocyclic scaffold.

**PAM2** and **PAM3** possess some structural dissimilarity. First, **PAM2** possesses two 2-hydroxyethoxy chains and two amide groups in the benzamide configuration while **PAM3** contains four of each. It is important to mention that the alcohol chains are positioned for synthetic ease. The synthesis and gelation properties of **PAM1** are reported in the literature [39]. **PAM1** was used in this study as a control molecule because of the absence of 2-hydroxyethoxy chains in its structure, providing a reliable comparison.

## Results and Discussion

**Scheme 1:**
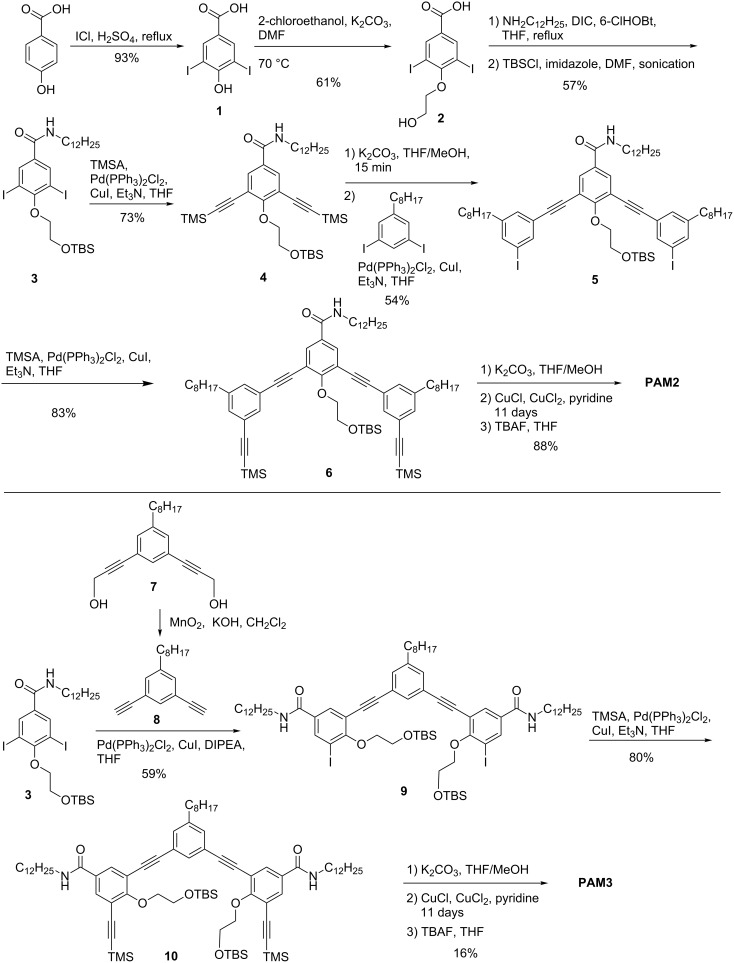
Synthetic pathway to **PAM2** and **PAM3**.

The synthetic pathway toward **PAM2** and **PAM3** is shown in Scheme 1. Starting from commercially available 4-hydroxybenzoic acid, iodination was performed to obtain compound **1** in excellent yield (93%). Then, 2-hydroxyethoxy chains were installed using K_2_CO_3_ to obtain compound **2** in good yield (61% yield). Amidation using diisopropylcarbodiimide (DIC) and 6-chlorohydroxybenzotriazole (6-ClHOBt) as coupling agents was then performed, followed by protection of the hydroxy moiety with TBS in order to facilitate the further synthetic steps by increasing the solubility of the compound (57% yield over 2 steps). Then, by using a standard Castro–Stephens–Sonogashira coupling, TMS protecting groups were installed (73% yield). After basic removal of the alkyne protective group, the half-macrocycle **5** was obtained by Castro–Stephens–Sonogashira coupling with previously reported 3,5-diiodooctylbenzene [39] in good yields despite of the possible polymerization side reaction (54% yield). TMS-protected alkynes were, then, installed on the half-macrocycle to afford compound **6** which can be deprotected using potassium hydroxide and directly used in modified Eglinton ring closing reaction in pseudo-high dilution to afford hydroxy-protected PAM. Deprotection was directly performed without purification using TBAF to afford **PAM2** in good yields (88% yield). Same approach was used for **PAM3**. Starting from previously synthesized compound **3**, the half-macrocycle **9** was obtained by standard Castro–Stephens–Sonogashira coupling with dialkyne **8** in good yield (59%). It is noteworthy that compound **8** was obtained from oxidative deprotection of compound **7**. After installing TMS-protected alkynes with good yields (compound **10**, 80% yield), selective removal of TMS using potassium hydroxide and ring closing reaction under modified Eglinton conditions were performed to afford a TBS protected PAM. Deprotection of TBS using TBAF was then realized without purification to afford **PAM3** in rather low yield. We hypothesized that this low yield can be due to the increased steric hindrance for compound **10** (2 OTBS groups) compared to compound **6** (1 OTBS group), impairing the macrocyclization reaction and leading to a significant amount of oligomerized product. It is noteworthy that purification of **PAM3** was realized by precipitation in methanol. Standard column chromatography was not effective due to the low solubility of the macrocycle after deprotection of the hydroxy-containing side chains.

The gelation properties of **PAM2** and **PAM3** have been studied and compared to those of **PAM1**. For each PAM, a precise quantity was mixed with the solvent in a capped glass tube. After sonication to breakdown the aggregates, the mixture was heated until a clear solution was obtained. Then, the solution was allowed to slowly cool at room temperature and the formation of the gel was confirmed in each case by the tube inversion method [39]. The gelation properties of **PAM1, PAM2** and **PAM3** in different solvents are summarized in Table S1 (Supporting Information File 1). **PAM2**, containing two amides and two 2-hydroxyethoxy side chains, has exactly the same gelation properties as **PAM1**, demonstrating the absence of the influence of the addition of side chains on the gelation properties. In the case of **PAM3**, however, most of the solvents tested did not lead to gel formation. Thus, only cyclohexane and toluene resulted in a partial gel state. The presence of four amide functions and four 2-hydroxyethoxy groups lead to a significant decrease of solubility, even in hot solvents, limiting the formation of organogels.

In order to investigate the crystallization temperature of solvents within the gel and to compare the influence of the 2-hydroxyethoxy side chains on the thermal stability of the gel, two gel samples were prepared with **PAM1** and **PAM2** in cyclohexane at a 10 mg/mL concentration and subjected to differential scanning calorimetry (DSC). The DSC analysis was carried out at temperatures ranging from 298 to 223 K. In both cases, a very sharp exotherm attributable to the crystallization of supercooled solvent was observed (see Figure S22 and S23 in Supporting Information File 1). For **PAM2**, the exotherm at 279 K is closer to the freezing point of cyclohexane (280 K) than for **PAM1**, which has an exotherm at 267 K. This result suggests that the gel of **PAM2** might be less organized than that of **PAM1**. By heating the gel from 223 to 298 K, an endotherm was observed in both cases, which is closely related to the melting point of free cyclohexane [39].

**Figure 2:**
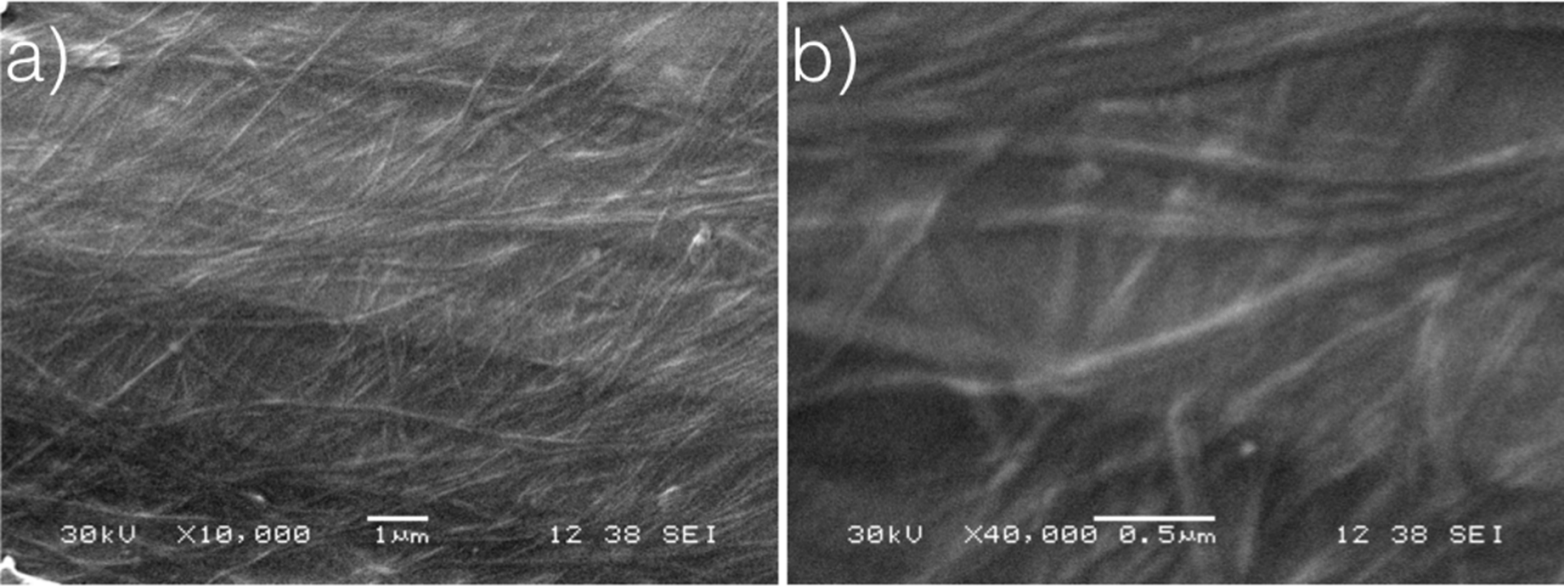
Scanning electron microscopy (SEM) images of **PAM2** xerogel in cyclohexane (10 mg/mL). Scales are a) 1 μm and b) 0.5 μm.

To explore the morphology of the structures created during the gel formation, scanning electron microscopy (SEM) was performed on **PAM2** only since **PAM3** did not present any gelation properties. For SEM analysis, a gel sample was allowed to dry at room temperature on a metallic substrate to form a xerogel. Then, gold was sputtered on the sample prior to imaging. SEM images of **PAM2** are shown in Figure 2. As previously observed with **PAM1** and other phenylacetylene macrocycles, a dense network of nanofibrils was formed during the gelation process [42–48]. These nanofibrils, commonly observed in the gel state, resulted from the strong intermolecular interactions that create long-range one-dimensional arrays of molecules [49–50].

In order to gain some insights on the molecular packing within the assembly, X-ray diffraction (XRD) was performed on **PAM2** (see Figure S21 in Supporting Information File 1). Unlike **PAM1**, the presence of a columnar arrangement cannot be established from the diffractogram [31,39]. Instead, only a broad peak at 2θ = 20° was observed, indicating the presence of intramolecular liquid-like order between the alkyl chains [2]. The absence of a well-defined pattern suggests that **PAM2** does not organize as well as **PAM1** in the gel state and that the 2-hydroxyethoxy chains are unfavourable for the long-range supramolecular organization.

Despite this unexpected finding, irradiation of a xerogel sample of **PAM2** in cyclohexane (10 mg/mL) was performed for 24 h under UV light (254 nm). The resulting blue material was purified using size-exclusion chromatography (SEC) (Bio-Beads SX-1) to remove all traces of the starting macrocycle. To our surprise, an increase of the polymerization yields was observed every time a polymerization was achieved with **PAM2**. In several attempts, a maximum yield of 30% (soluble material) was obtained after purification, representing a two-fold increase compared to the best result obtained for the same PAM scaffold (≈15% yield). This result suggests that the diyne units in **PAM2** are in a more suitable orientation than **PAM1** to undergo a topochemical polymerization, although gelation properties and XRD result seem to suggest otherwise. It is also possible that the apparent yield increase comes from a better solubility of the PDA obtained from **PAM2** compared to that obtained from **PAM1**, although quantitative measurements of the solubility have not been conducted because of the small quantity of materials prepared.

**Figure 3:**
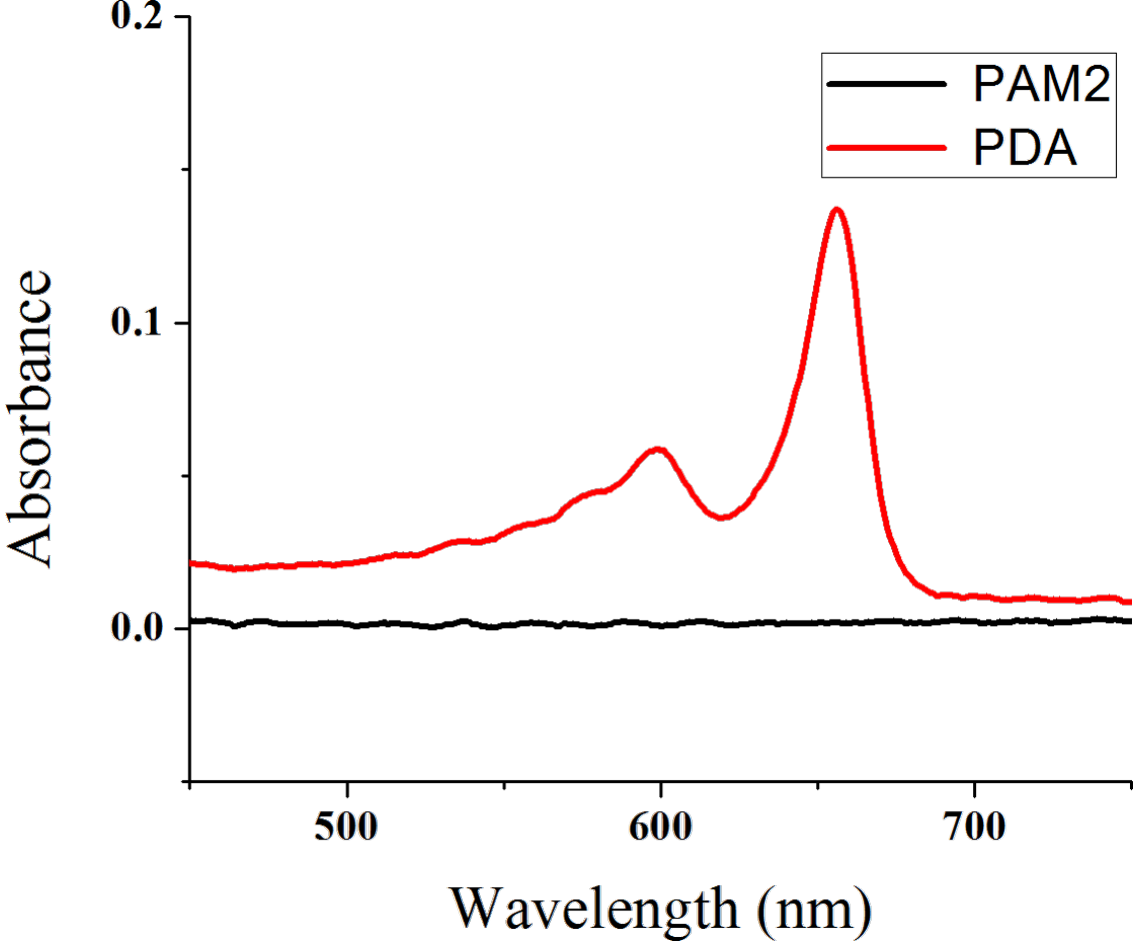
UV–vis spectrum of **PAM2** before (black) and after (red) polymerization (PDA).

**Figure 4:**
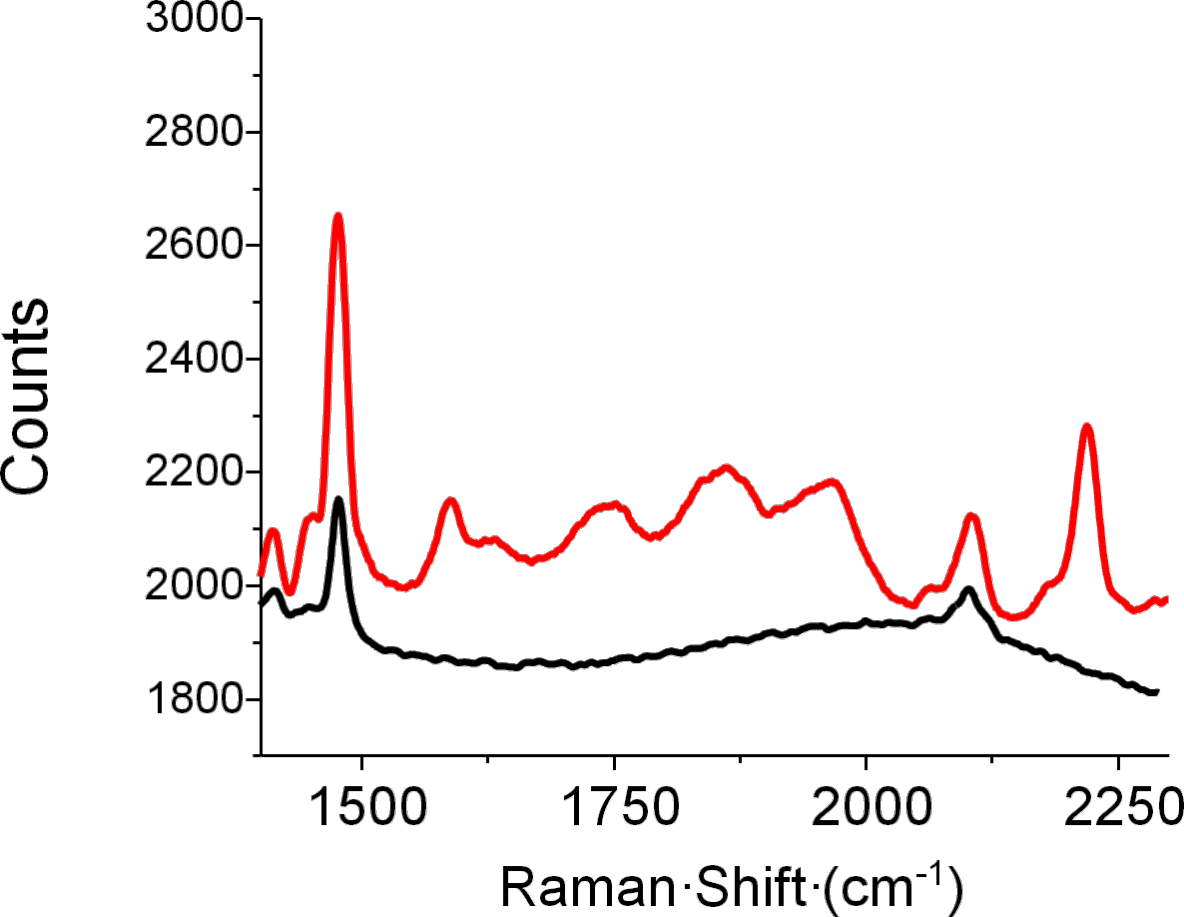
Background-corrected Raman spectra of **PAM2** (red) and the blue material obtained after UV irradiation (black).

Given the observed polymerization of diyne units, UV–vis spectroscopy was performed to confirm the appearance of polydiacetylene. As shown in Figure 3, the UV–vis spectra shows absorption bands at 600 and 650 nm, associated with the red and blue bands of the PDA chain, respectively [51]. To determine whether all the diyne units of **PAM2** reacted during irradiation, Raman spectroscopy was performed on the resulting PDA (Figure 4). As expected, no band associated with the diyne unit (generally around 2200 cm^−1^) is present. The final PDA presented bands at 1470 and 2100 cm^−1^, which could be respectively associated to alkene and alkyne moieties of PDA. It should be noted that these two bands are also present in the Raman spectrum of **PAM2** due to partial photo-induced polymerization during spectrum acquisition. This demonstrates the high reactivity of **PAM2** under UV irradiation. Further research on the resulting nanoarchitectures is currently underway to establish if covalent nanotubes have been formed and to study their properties.

To assess the presence of nanotubes or nanorods, HRTEM analysis has been performed on the purified blue materials. Although one-dimensional features can be observed at different locations on the grid (see Figure S24 in Supporting Information File 1), the images were not clear enough to confirm whether or not **PAM2** has been transformed exclusively into nanotubes or nanorods and attempts to disperse individual molecules in organic solvents failed. Considering the strict structural parameters needed to undergo a topochemical polymerization and the one-dimensional nature of the starting xerogel of **PAM2**, it is unlikely – but not impossible – that the final blue materials can be made of other nanoarchitectures than the nanotubular or nanorods one. Nonetheless, no conclusive remarks can be made and more analysis will have to be performed in order to gain better insight about the final structure of the PDA obtain from **PAM2**.

## Conclusion

In summary, the synthesis and self-assembly of two new phenylacetylene macrocycles were performed. To improve the self-assembly properties in the gel state and further yield to better topochemical polymerization yield, the incorporation of 2-hydroxyethoxy side chains was accomplished. The gelation properties demonstrated that the incorporation of two of these chains (**PAM2**) did not affect the gelation properties while the incorporation of four side chains and four amide groups significantly alter the solubility of the resulting PAM (**PAM3**). Topochemical polymerization on self-assembled **PAM2** to yield PDA-based materials proved to be much more efficient than for the 2-hydroxyethoxy-free analogues (**PAM1**). Characterization is currently underway to investigate the precise nature of the nanoarchitectures formed.

## Supporting Information

File 1 Experimental part.
