# Supplementary material for: Improving the reactivity of phenylacetylene macrocycles toward topochemical polymerization by side chains modification
Source: Beilstein J Org Chem. 2014 Jul 15;10:1613–9. doi: 10.3762/bjoc.10.167 (PMC4142893; doi:10.3762/bjoc.10.167)
Supplement: File 1 — Experimental part. [file Beilstein_J_Org_Chem-10-1613-s001.pdf]

Supporting Information

for

Improving the reactivity of phenylacetylene macrocycles toward topochemical polymerization by side chains modification

Simon Rondeau-Gagné‡, Jules Roméo Néabo‡, Maxime Daigle, Katy Cantin and Jean-François Morin*

Address: Département de Chimie and Centre de recherche sur les Matériaux Avancés (CERMA), Université Laval, 1045 avenue de la Médecine, G1V 0A6 Québec, Québec, Canada

‡These authors have contributed equally to this work

Email: Jean-François Morin - jean-francois.morin@chm.ulaval.ca

*Corresponding author

Experimental part

Table of content

1- General remarks
2- Experimental procedures and data
3- ^{1}H and ^{13}C NMR spectra
4- Gelation properties of the new PAMs series
5- X-ray diffraction spectra (PXRD)
6- Differential scanning calorimetry (DSC)
7- Transmission electron microscopy (TEM)
8- References

1- General remarks

Chemical reagents were purchased from Sigma-Aldrich Co. Canada, Alfa Aesar Co., TCI America Co. or Oakwood Products Inc. and were used as received. Solvents used for organic synthesis were obtained from Fisher Scientific (except THF from Sigma- Aldrich Co. Canada) and purified with a Solvent Purifier System (SPS) (Vacuum Atmosphere Co., Hawthorne, USA). Other solvents were obtained from Fisher Scientific and were used as received. Tetrahydrofuran (THF) and triethylamine (Et_3N) used for Castro-Stephens-Sonogashira reactions were degassed 30 minutes prior to use. All anhydrous and air sensitive reactions were performed in oven-dried glassware under positive argon pressure. Analytical thin-layer chromatographies were performed with silica gel 60 F254, 0.25 mm pre-coated TLC plates (Silicycle, Québec, Canada). Compounds were visualized using 254 nm and/or 365 nm UV wavelength and/or aqueous sulfuric acid solution of ammonium heptamolybdate tetrahydrate (10 g/100 mL H_2SO_4 + 900 mL H_2O). Flash column chromatographies were performed on 230-400 mesh silica gel R10030B (Silicycle, Québec, Canada). Nuclear magnetic resonance (NMR) spectra were recorded on a Varian Inova AS400 spectrometer (Varian, Palo Alto, USA) at 400 MHz (^1H) and 100 MHz (^{13}C). High-resolution mass spectra (HRMS) were recorded with an Agilent 6210 Time-of-Flight (TOF) LC-MS apparatus equipped with an ESI or APPI ion source (Agilent Technologies, Toronto, Canada). UV-visible absorption spectra were recorded on a Varian diode-array spectrophotometer (model Cary 500) using 3-mm path length quartz cells. Scanning electron microscopy (SEM) images were taken using a

JEOL JSM-6360 LV. X-ray diffraction was recorded on Siemens X-rays Diffractometer (Model S3 D5000). DSC measurements were done on a Mettler Toledo (DSC 823e).

Gelation test

To test the gelation properties of PAMs in a given solvent, we proceeded as follow: in a vial, a PAMs was dissolved in a solvent. After dissolution by sonication, the vial was sealed and heated until a clear solution was obtained. The clear solution was allowed to slowly cool down at room temperature. The stability of the gel was confirmed by tube inversion.

SEM imaging

Organogel obtained in cyclohexane was deposited on a stainless steel substrate and allowed to dry for 3-4 days. Then, gold particles were sputtered on dried gel prior to imaging.

2- Experimental procedures and datas

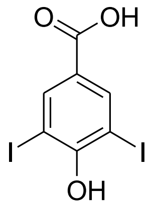

Compound 1. A round-bottom flask equipped with a magnetic stir bar is charged with 4-hydroxybenzoic acid (5.00 g, 36.2 mmol), 10 % H_2SO_4 aqueous solution (180 mL) and Iodine monochloride (17.6 g, 108 mmol) at 0°C. Then, the reaction mixture is heated to 80°C and stirred overnight. The precipitated mixture is filtered and washed with sodium

bisulfite and water to afford compound **1** (13.1 g, 93% yield) as a white amorphous powder. NMR ^{1}H (400 MHz, DMSO-d$_6$): 8.30 (s, 1H); 8.14 (s, 2H). NMR ^{13}C (100 MHz, DMSO-d$_6$): 165.1; 146.6; 140.8; 137.5; 86.6; HRMS was not possible due to low ionization of compound **1** by APPI-TOF and ESI-TOF mass spectroscopy.

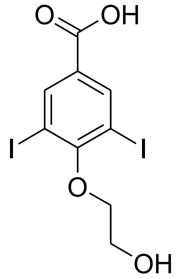

Compound 2. A round bottom flask equipped with a magnetic stir bar is charged with compound **1** (14.0 g, 35.9 mmol), water (72 mL), NaOH (5.02 g, 125 mmol) and 2-chloroethanol (8.67 g, 108 mmol). The reaction mixture is heated to 82°C and KI (596 mg, 3.59 mmol) is added. The reaction is then stirred overnight and allowed to cool to rt. The mixture is acidified with HCl to pH=5 and the solid is filtered under vacuo. The filtrate is washed with MeOH, EtOAc and with an hexanes in EtOAc 1:1 mixture to afford compound **2** (9.54 g, 61% yield) as a white amorphous powder. ^{1}H NMR (400 MHz, DMSO-d$_6$): 8.28 (s, 2H), 3.96 (t, J = 5.1 Hz, 2H), 3.83 (t, J = 5.1 Hz, 2H); ^{13}C NMR (100 MHz, DMSO-d$_6$): 164.8; 161.8; 140.9; 130.5; 92.4; 74.9; 60.5; HRMS (ESI-TOF) m/z calcd for $C_9H_8I_2O_4[M+H]^+$: 434.8585, found 434.8566.

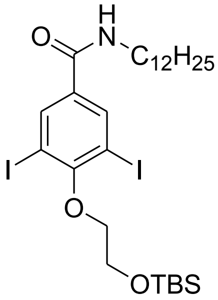

Compound 3. A round bottom flask equipped with a magnetic stir bar is charged with compound **2** (4.50 g, 11.6 mmol), 6-ClHOBt (1.78 g, 10.5 mmol), DIC (1.47 g, 11.6 mmol), dodecylamine (2.24 g, 12.8 mmol) and THF (58 mL). The reaction mixture is stirred overnight and allowed to cool down. The mixture is then diluted with EtOAc and the organic layer is wash with water (3x), brine and dried with Na_2SO_4. The solvents are removed under reduced pressure and the crude product is directly charged without further purification in a round bottom flask equipped with a magnetic stir. Imidazole (1.84 g, 27.2 mmol), TBSCl (1.96 g, 12.9 mmol) and DMF (10 mL) are then added and the mixture is stirred for 1h in an ultrasonic bath. The mixture is then diluted with EtOAc and the organic layer is washed with water (3x), brine and dried with Na_2SO_4. The crude product is then purified by flash chromatography on silica gel using 10% hexanes in acetone as eluant to afford compound **3** (3.41 g, 57% yield, 2 steps) as a white amorphous powder. ^{1}H NMR (400 MHz, $CDCl_3$): 8.13 (s, 2H), 6.13 (m, 1H), 4.05 (m, 4H), 3.39 (q, J = 6.7 Hz, 2H), 1.58 (m, 2H), 1.24 (s, 18H), 0.92 (s, 9H), 0.87 (t, J = 6.6 Hz, 3H), 0.13 (s, 6H); ^{13}C NMR (100 MHz, $CDCl_3$): 164.0; 160.1; 138.6; 134.0; 90.8; 73.9; 62.2; 40.4; 31.9; 29.6 (3C); 29.5 (2C); 29.3 (2C); 26.9; 25.9; 22.7; 18.4; 14.1; -5.1; HRMS (APPI-TOF) *m/z* calcd for $C_{27}H_{47}I_2NO_3Si[M+H]^+$: 717.1515, found 717.153.

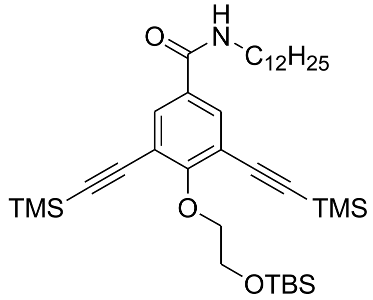

Compound 4. A round bottom flask equipped with a magnetic stir bar is charged under nitrogen with compound **3** (2.00 g, 2.79 mmol), Et_3N (1.5 mL), THF (14 mL), $PdCl_2(PPh_3)_2$ (78 mg, 0.11 mmol) and CuI (21 mg, 0.11 mmol). TMSA (1.09 g, 11.2 mmol) is then added and the mixture is stirred overnight. The reaction is then diluted with CH_2Cl_2 and the organic layer is washed with NH_4Cl, water and brine. The organic layer is then dried with Na_2SO_4 and the solvent are removed under reduced pressure. The crude product is purified by flash chromatography on silica gel using hexanes to 4% hexanes in acetone as eluents to afford compound **4** (1.34 g, 73% yield) as light orange oil. ^{1}H NMR (400 MHz, $CDCl_3$): 7.77 (s, 2H), 6.21 (m, 1H), 4.36 (t, J = 5.9 Hz, 2H), 3.98 (t, J = 5.9 Hz, 2H), 3.39 (q, J = 6.5 Hz, 2H), 1.57 (m, 2H), 1.26 (m, 18H), 0.90 (m, 12H), 0.24 (s, 18H), 0.07 (s, 6H); ^{13}C NMR (100 MHz, $CDCl_3$): 165.5; 163.9; 132.6; 129.6; 117.3; 100.2; 99.9; 74.5; 62.5; 40.2; 34.6; 31.9; 31.6; 29.6 ; 29.5; 29.3; 26.9; 25.8; 25.7; 25.3; 22.6; 20.7; 18.3; 17.9; 14.1; HRMS (APPI-TOF) *m/z* calcd for $C_{37}H_{65}NO_3Si_3[M+H]^+$: 656.4345, found 656.4362.

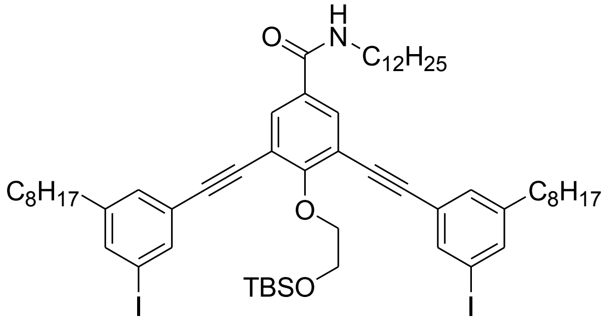

Compound 5. A round bottom flask equipped with a magnetic stir bar is charged with compound **4** (3.00 g, 2.78 mmol), K_2CO_3 (767 mg, 5.55 mmol), THF (14 mL) and MeOH (14 mL). The reaction is stirred until complete disappearance of the starting product by TLC. The mixture is then diluted with CH_2Cl_2 and washed with water. The organic layer is then dried with Na_2SO_4 and the solvent are removed under reduced pressure. The crude product is used directly with further purification and is charged in a round bottom flask equipped with a magnetic stir bar under nitrogen with 3,5-diiodooctylbenzene (3.33 g, 7.53 mmol) and Et_3N (25 mL). $PdCl_2(PPh_3)_2$ (71 mg, 0.10 mmol) and CuI (19 mg, 0.10 mmol) are then added and the mixture is stirred overnight. The mixture is diluted with CH_2Cl_2 and the organic layer is washed with NH_4Cl, water, brine and dried with Na_2SO_4. The solvent is removed under reduced pressure and the crude product is purified by flash chromatography on silica gel using hexanes to 6% hexanes in acetone as eluents to afford compound **5** (1.55 g, 54% yield) as light orange oil. ^{1}H NMR (400 MHz, $CDCl_3$): 7.84 (s, 2H), 7.69 (s, 2H), 7.53 (s, 2H), 7.30 (s, 2H), 6.02 (m, 1H), 4.45 (t, J = 4.8 Hz, 2H), 4.05 (t, J = 4.8 Hz, 2H), 3.44 (q, J = 6.6 Hz, 2H), 2.54 (t, J = 7.4 Hz, 4H), 1.59 (m, 12H), 1.28 (m, 32H), 0.88 (m, 9H), 0.85 (m, 15H); ^{13}C NMR (100 MHz, $CDCl_3$): 165.4; 163.2; 145.3; 137.9; 137.3; 132.2; 130.9; 130.0; 124.6; 117.3; 93.4; 93.2; 85.5; 75.1; 62.7; 40.3; 35.4; 31.9; 31.8; 31.2; 29.6 (4C); 29.5; 29.4; 29.3 (2C); 29.2 (2C); 27.0; 25.9 (2C); 22.7;

22.6; 18.3; 14.1 (2C): HRMS (APPI-TOF) *m/z* calcd for $C_{59}H_{87}I_2NO_3Si[M+H]^+$: 1142.4668, found 1142.4708.

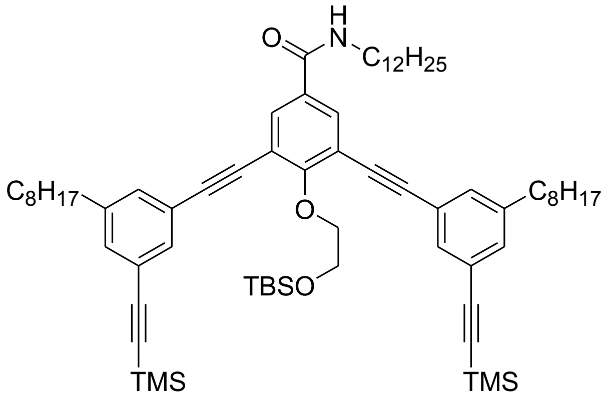

Compound 6. A round bottom flask equipped with a magnetic stir bar is charged under nitrogen with compound **5** (380 mg, 0.33 mmol), Et_3N (3 mL), THF (3 mL), $PdCl_2(PPh_3)_2$ (9 mg, 0.01 mmol) and CuI (3 mg, 0.01 mmol). TMSA (98 mg, 1.00 mmol) is then added and the mixture is stirred overnight. The reaction is then diluted with CH_2Cl_2 and the organic layer is washed with NH_4Cl, water and brine. The organic layer is dried with Na_2SO_4 and the solvent are removed under reduced pressure. The crude product is purified by flash chromatography on silica gel using hexanes to 4% hexanes in acetone as eluents to afford compound **6** (243 mg, 83% yield) as light orange oil. ^1H NMR (400 MHz, $CDCl_3$): 7.84 (s, 2H), 7.47 (s, 2H), 7.30 (s, 2H), 7.29 (s, 2H), 6.04 (m, 1H), 4.46 (t, *J* = 5.8 Hz, 2H), 4.07 (t, *J* = 5.8 Hz, 2H), 3.45 (q, *J* = 6 Hz, 2H), 2.57 (t, *J* = 7.4 Hz, 4H), 1.61 (m, 12H), 1.28 (m, 32H), 0.88 (m, 9H), 0.85 (s, 15H), 0.26 (s, 18H); ^{13}C NMR (100 MHz, $CDCl_3$): 163.1; 143.4; 132.3 (2C), 132.2; 131.7; 129.9; 123.3; 122.9; 117.6; 104.3; 94.6; 94.1; 84.8; 75.0; 62.7; 40.2; 35.5; 31.9 (2C); 31.2; 29.7 (2C); 29.6 (2C); 29.5; 29.4; 29.3 (3C); 29.2 (2C); 29.1; 26.9; 25.9 (2C); 22.7; 22.6; 18.4; 14.1;

0.08; HRMS (APPI-TOF) *m/z* calcd for $C_{69}H_{105}NO_3Si_3[M+H]^+$: 1080.7475, found 1080.7531.

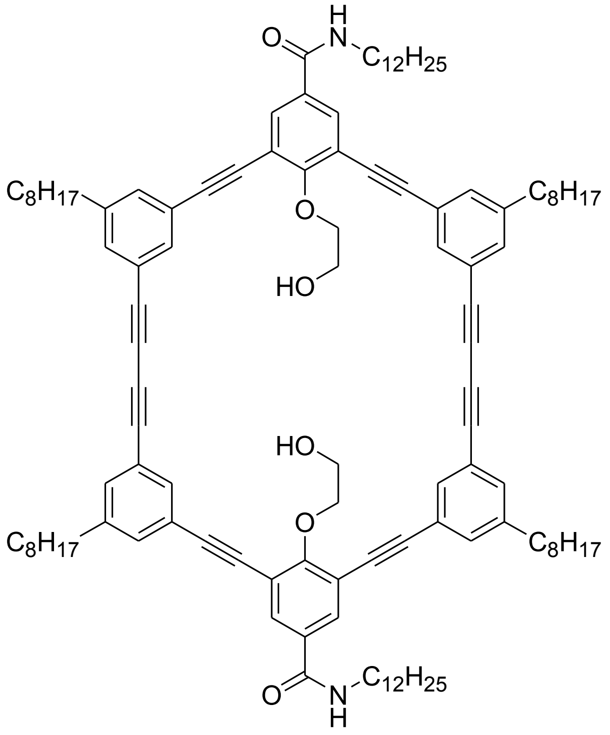

PAM2. . A round bottom flask equipped with a magnetic stir bar was charged with compound **6** (270 mg, 0.25 mmol), THF (1.2 mL), MeOH (1.2 mL) and K_2CO_3 (69 mg, 0.50 mmol). The reaction mixture was stirred until complete disappearance of the starting product by TLC, diluted with CH_2Cl_2, washed with water (3x), dried with sodium sulfate and the solvent was removed under reduced pressure. The resulting product was charged without further purification in a round bottom flask equipped with a magnetic stir bar with degassed pyridine (13 mL). Another round bottom flask equipped with a magnetic stir bar was charged with CuCl (1.88 g, 18.9 mmol), $CuCl_2$ (395 mg, 2.93 mmol) and degassed pyridine (38 mL) under N_2 atmosphere. The first solution was added dropwise

to the catalyst solution over 4 days using a syringe pump and the reaction mixture was stirred for an additional 7 days. The reaction mixture was diluted with $CHCl_3$ and poured in water. The organic layer was extracted successively with water, 25% aqueous NH_4OH, water, 10% aqueous acetic acid, water, 10% aqueous NaOH and brine. The organic layer was dried with sodium sulfate and the solvent was removed under reduced pressure. A round bottom flask equipped with a magnetic stir bar was then charged with the crude product (without purification), TBAF 1.0 M solution in THF (0.51 mL, 0.51 mmol) and THF (1.7 mL). The reaction was stirred until complete disappearance of the starting product by TLC. The reaction mixture was then concentrated under reduced pressure and poured in MeOH. The precipitate was filtered under vacuo and the solid was dissolved. The crude product purified by flash chromatography on silica gel using 20% hexanes in acetone as eluent to afford **PAM2** (123 mg, 88% yield) as a white amorphous powder. ^{1}H (400 MHz, $CDCl_3$): 7.78 (s, 2H), 7.55 (s, 4H), 7.46 (s, 4H), 7.28 (s, 2H), 7.06 (br s, 8H), 6.96 (m, 2H), 4.55 (t, J = 4.8 Hz, 4H), 4.16 (br s, 2H), 3.96 (m, 4H), 3.49 (m, 4H), 2.52 (t, J = 7.6 Hz, 8H), 1.73 (m, 4H), 1.60 (m, 8H), 1.29 (m, 78H), 0.88 (m, 16H); ^{13}C NMR: Due to the poor solubility of **PAM2** in common organic solvent, acquisition of clean spectrum was not afforded; HRMS (APPI-TOF) *m/z* calcd for $C_{114}H_{146}N_2O_6[M+H]^+$: 1640.1254, found 1640.1180.

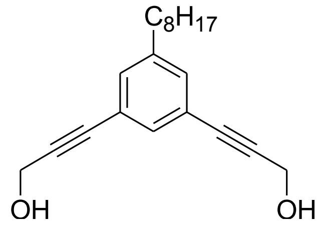

Compound 7. A round bottom flask equipped with a magnetic stir bar is charged under nitrogen with 3,5-diiodooctylbenzene (1.00 g, 2.26 mmol), Et_3N (1.2 mL, 9.05 mmol), THF (22 mL), $PdCl_2(PPh_3)_2$ (64 mg, 0.09 mmol) and CuI (17 mg, 0.09 mmol). Propargylic alcohol (507 mg, 9.05 mmol) is then added and the mixture is stirred overnight. The reaction is then diluted with CH_2Cl_2 and the organic layer is washed with NH_4Cl, water and brine. The organic layer is then dried with Na_2SO_4 and the solvent are removed under reduced pressure. The crude product is purified by flash chromatography on silica gel using 5% CH_2Cl_2 in acetone as eluant to afford compound **7** (598 mg, 89% yield) as light orange oil. ^{1}H NMR (400 MHz, $CDCl_3$): 7.29 (s, 1H), 7.19 (s, 2H), 4.47 (s, 4H), 2.74 (s, 2H), 2.49 (t, J = 7.6 Hz, 2H), 1.53 (t, J = 6.2 Hz, 2H), 1.25 (br s, 9H), 0.87 (t, J = 6.7 Hz, 3H); ^{13}C NMR (100 MHz, $CDCl_3$): 132.0; 131.9; 122.6; 85.09; 51.7; 35.4; 31.9; 31.1; 29.4; 29.2; 29.1; 22.6; 14.1; HRMS was not possible due to low ionization of compound **9** by APPI-TOF and ESI-TOF mass spectroscopy.

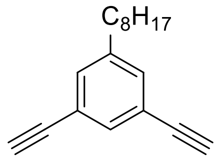

Compound 8. A round bottom flask equipped with a magnetic stir bar is charged with compound **7** (600 mg, 2.01 mmol), KOH (1.81 g, 32.2 mmol), MnO_2 (2.79 g, 32.2 mmol) and CH_2Cl_2 (10 mL). The reaction is stirred until complete disappearance of the starting product by TLC. The mixture is then diluted with CH_2Cl_2 and washed with water. The

organic layer is then filtered on celite, dried with Na_2SO_4 and the solvent are removed under reduced pressure. The crude product was directly used with further purification.

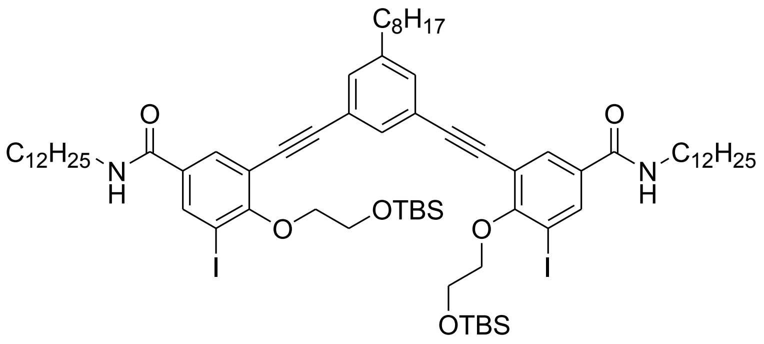

Compound 9. A round bottom flask equipped with a magnetic stir bar is charged with compound **8** (crude mixture, 1 eq.), compound **3** (901 mg, 1.26 mmol), DIPEA (0.59 mL, 3.36 mmol) and THF (4 mL). $PdCl_2(PPh_3)_2$ (12 mg, 0.02 mmol) and CuI (3 mg, 0.02 mmol) and the mixture is stirred overnight. The mixture is diluted with CH_2Cl_2 and the organic layer is washed with NH_4Cl, water, brine and dried with Na_2SO_4. The solvent is removed under reduced pressure and the crude product is purified by flash chromatography on silica gel using hexanes to 8% hexanes in acetone as eluents to afford compound **9** (351 mg, 59% yield) as light orange oil. ^{1}H NMR (400 MHz, $CDCl_3$): 8.17 (s, 1H), 8.15 (s, 1H), 7.88 (s, 1H), 7.86 (s, 1H), 7.53 (m, 1H), 7.37 (s, 1H), 7.35 (s, 1H), 6.01 (m, 2H), 4.36 (t, J = 4.5 Hz, 4H), 4.09 (t, J = 4.5 Hz, 4H), 3.44 (q, J = 6 Hz, 4H), 2.62 (m, 2H), 1.61 (m, 8H), 1.26 (m, 61H), 0.88 (m, 30H): ^{13}C NMR (100 MHz, $CDCl_3$): 164.8; 161.8; 138.6; 138.3; 134.0; 132.5; 132.0; 131.8 131.7; 122.9; 117.4; 94.6; 92.4; 90.8; 74.9; 62.6; 62.3; 40.3; 31.9 (2C); 31.2; 29.7; 29.6 (3C); 29.5; 29.4 (2C); 29.3 (2C);

29.2; 26.9; 25.9 (2C); 25.8; 22.7 (2C); 18.4; 14.1: HRMS (APPI-TOF) *m/z* calcd for $C_{72}H_{114}I_2N_2O_6Si_2[M+H]^+$: 1413.6378, found 1413.6473.

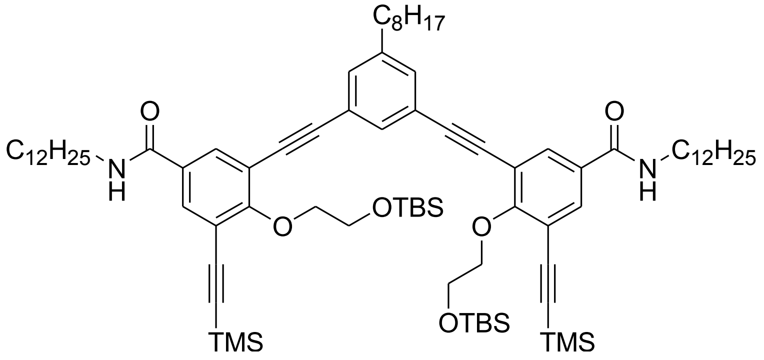

Compound 10. A round bottom flask equipped with a magnetic stir bar is charged under nitrogen with compound **9** (1.60 g, 1.13 mmol), Et_3N (0.62 mL, 4.53 mmol), THF (11 mL), $PdCl_2(PPh_3)_2$ (40 mg, 0.06 mmol) and CuI (11 mg, 0.06 mmol). TMSA (445 mg, 4.53 mmol) is then added and the mixture is stirred overnight. The reaction is then diluted with CH_2Cl_2 and the organic layer is washed with NH_4Cl, water and brine. The organic layer is dried with Na_2SO_4 and the solvent are removed under reduced pressure. The crude product is purified by flash chromatography on silica gel using hexanes to 5% hexanes in acetone as eluents to afford compound **10** (1.22 g, 80% yield) as light orange oil. ^1H NMR (400 MHz, $CDCl_3$): 7.84 (s, 1H), 7.81 (s, 2H), 7.75 (s, 2H), 7.31 (s, 2H), 6.02 (m, 2H), 4.40 (t, *J* = 5.7 Hz, 2H), 3.99 (t, *J* = 5.7 Hz, 2H), 3.39 (q, *J* = 6.9 Hz, 4H), 2.57 (m, 2H), 1.58 (m, 8H), 1.22 (br s, 62H), 0.83 (m, 30H), 0.24 (s, 18H): ^{13}C NMR (100 MHz, $CDCl_3$): 163.5; 143.6; 132.5; 132.3; 131.8; 129.8; 123.1; 117.4; 117.3; 100.4; 100.0; 93.9; 93.8;84.9; 74.8; 62.7; 62.6; 47.9; 40.2 (2C); 35.6; 35.5; 31.8 (3C); 31.2; 29.6

(3C); 29.5 (2C); 29.4 (2C); 29.3 (2C); 29.2 (3C); 29.1; 29.0; 26.9; 25.8; 22.6; 18.3; 14.1; 0.22: HRMS (APPI-TOF) *m/z* calcd for $C_{82}H_{132}N_2O_6Si_4[M+H]^+$: 1353.9235, found 1353.9329.

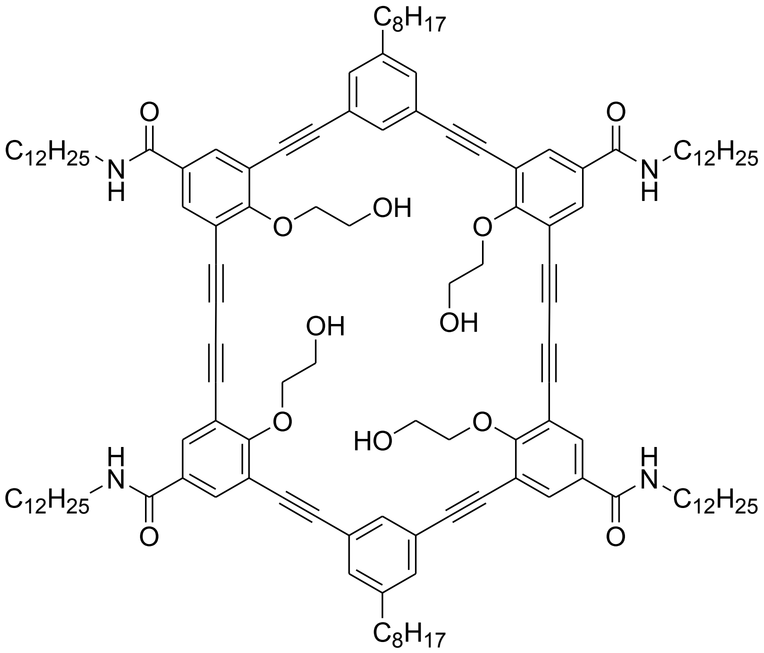

PAM3. A round bottom flask equipped with a magnetic stir bar was charged with compound **10** (380 mg, 0.28 mmol), THF (1.4 mL), MeOH (1.4 mL) and K_2CO_3 (9 mg, 0.07 mmol). The reaction mixture was stirred until complete disappearance of the starting product by TLC, diluted with CH_2Cl_2, washed with water (3x), dried with sodium sulfate and the solvent was removed under reduced pressure. The resulting product was charged without further purification in a round bottom flask equipped with a magnetic stir bar with TBAF 1.0 M solution in THF (1.12 mL, 1.12 mmol) and THF (4.2 mL). The reaction mixture was stirred until complete disappearance of the starting product by TLC, diluted with CH_2Cl_2, washed with water (3x), dried with sodium sulfate and the solvent was removed under reduced pressure. The resulting product was charged without further purification in a round bottom flask equipped with a magnetic stir bar with degassed

pyridine (5 mL). Another round bottom flask equipped with a magnetic stir bar was charged with CuCl (1.14 g, 11.6 mmol), $CuCl_2$ (241 mg, 1.79 mmol) and degassed pyridine (23 mL) under N_2 atmosphere. The first solution was added dropwise to the catalyst solution over 4 days using a syringe pump and the reaction mixture was stirred for an additional 7 days. The reaction mixture was diluted with $CHCl_3$ and poured in water. The organic layer was extracted successively with water, 25% aqueous NH_4OH, water, 10% aqueous acetic acid, water, 10% aqueous NaOH and brine. The organic layer was dried with sodium sulfate and the solvent was removed under reduced pressure. The crude product was purified by flash chromatography on silica gel using $CHCl_3$ to 5% acetone in $CHCl_3$ as eluents to afford **PAM3** (45 mg, 16 % yield over 3 steps) as a white amorphous powder. ^{1}H NMR and ^{13}C NMR: Due to the poor solubility of **PAM3** in common organic solvent, acquisition of clean spectrum was not afforded. The macrocycle shown strong aggregation by NMR even at 100°C in deuterated DMSO. HRMS (APPI-TOF) *m/z* calcd for $C_{128}H_{172}N_4O_{12}[M+H]^+$: 1958.3045, found 1958.3041.

3- ^{1}H and ^{13}C NMR spectrum

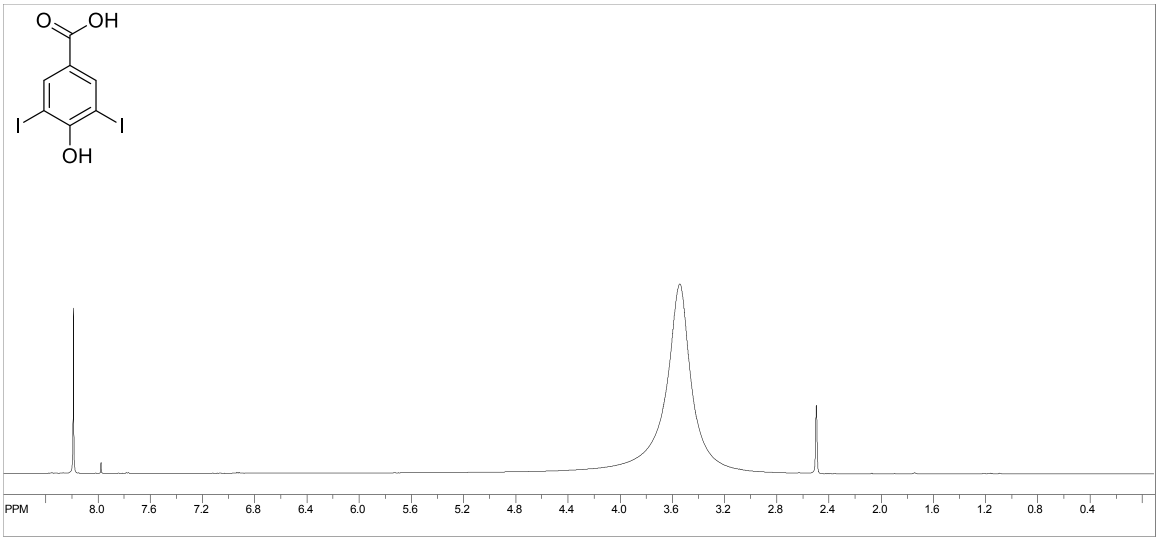

Figure S1. ^{1}H NMR of compound **1** in DMSO-d_6.

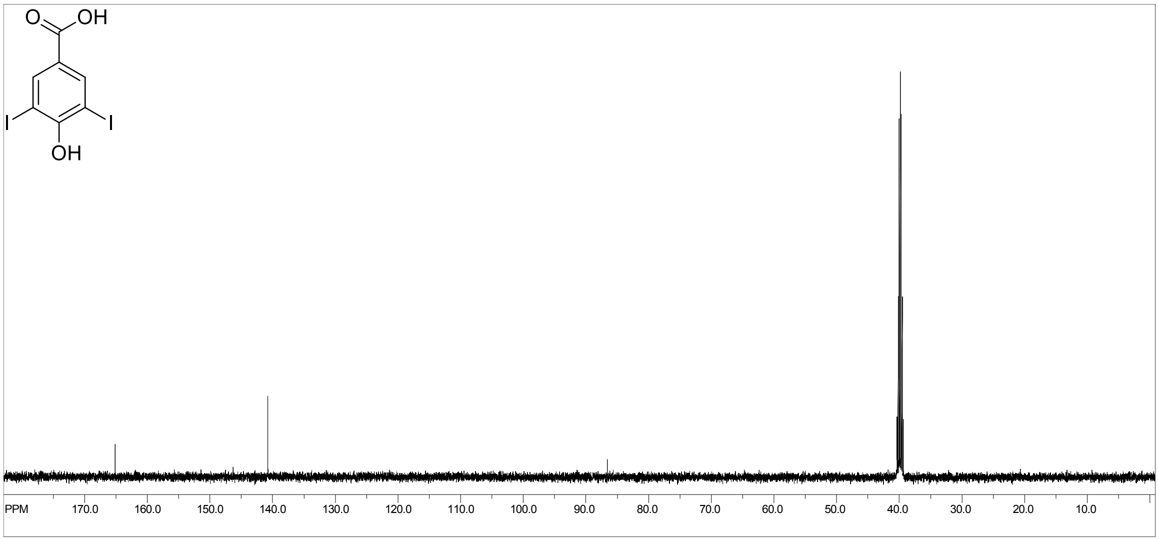

Figure S2. ^{13}C NMR of compound **1** in DMSO-d_6.

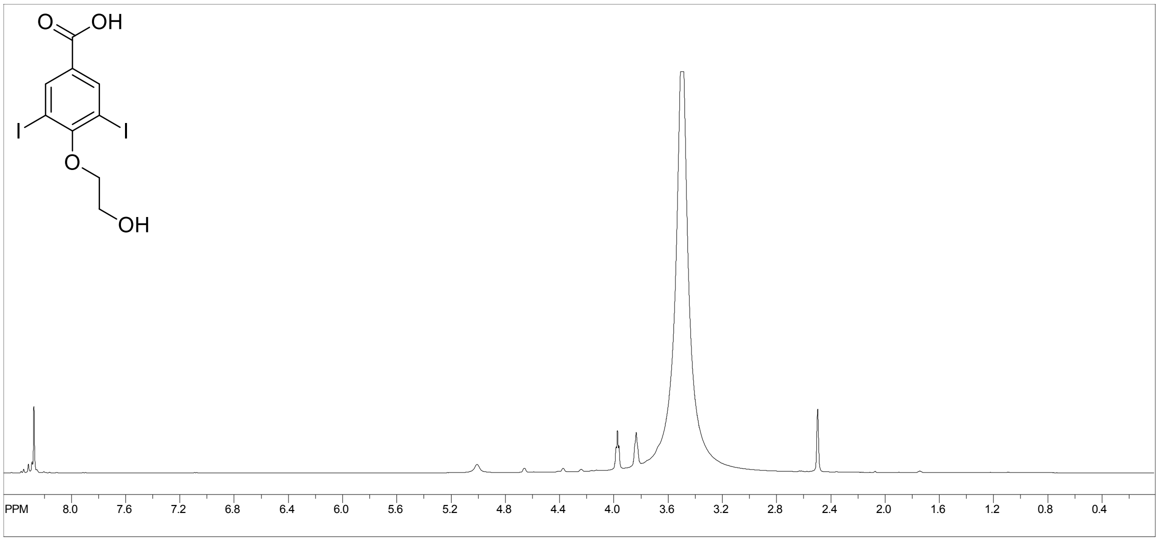

Figure S3. ^{1}H NMR of compound **2** in DMSO-d_6

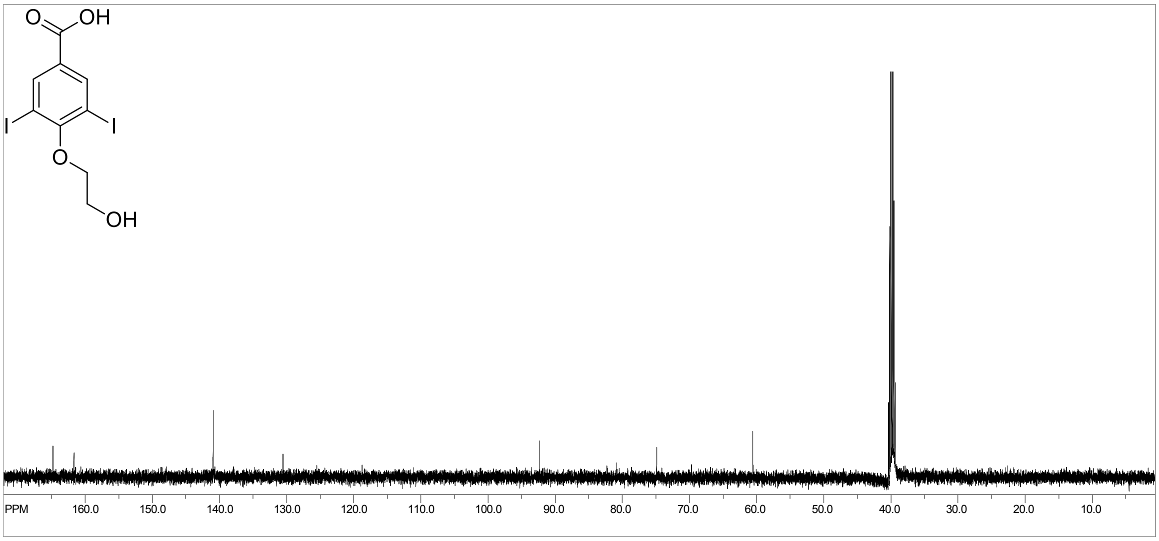

Figure S4. ^{13}C NMR of compound **2** in DMSO-d_6

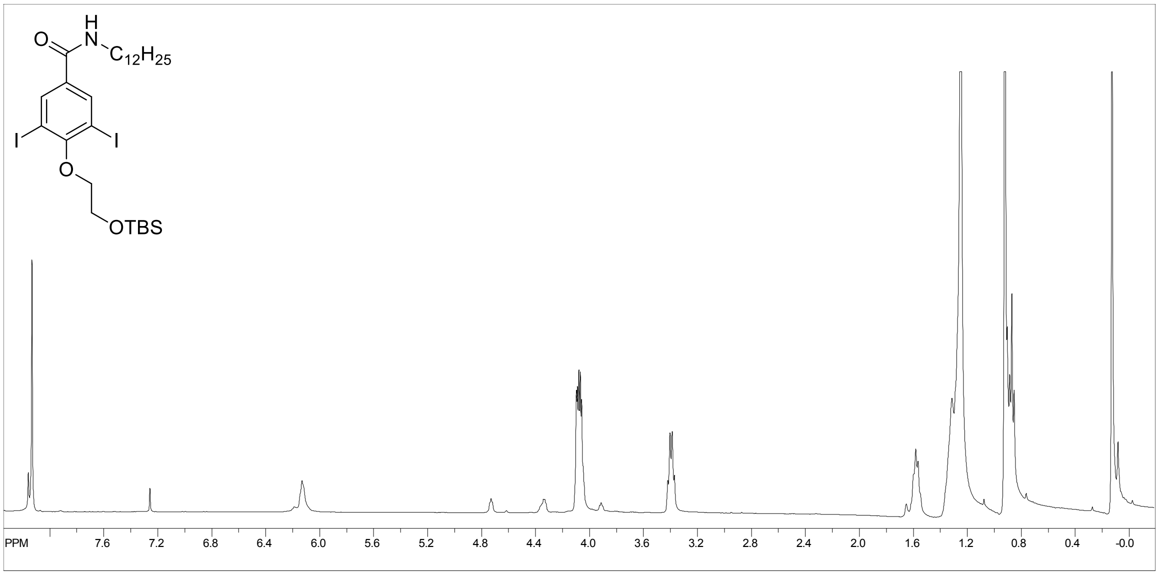

Figure S5. ^{1}H NMR of compound **3** in $CDCl_3$

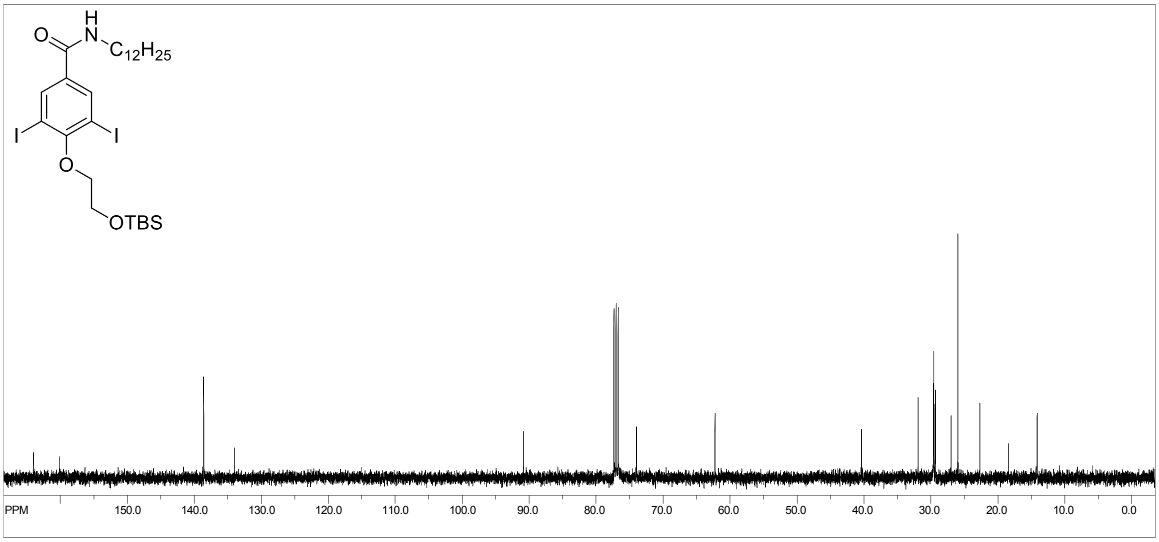

Figure S6. ^{13}C NMR of compound **3** in $CDCl_3$.

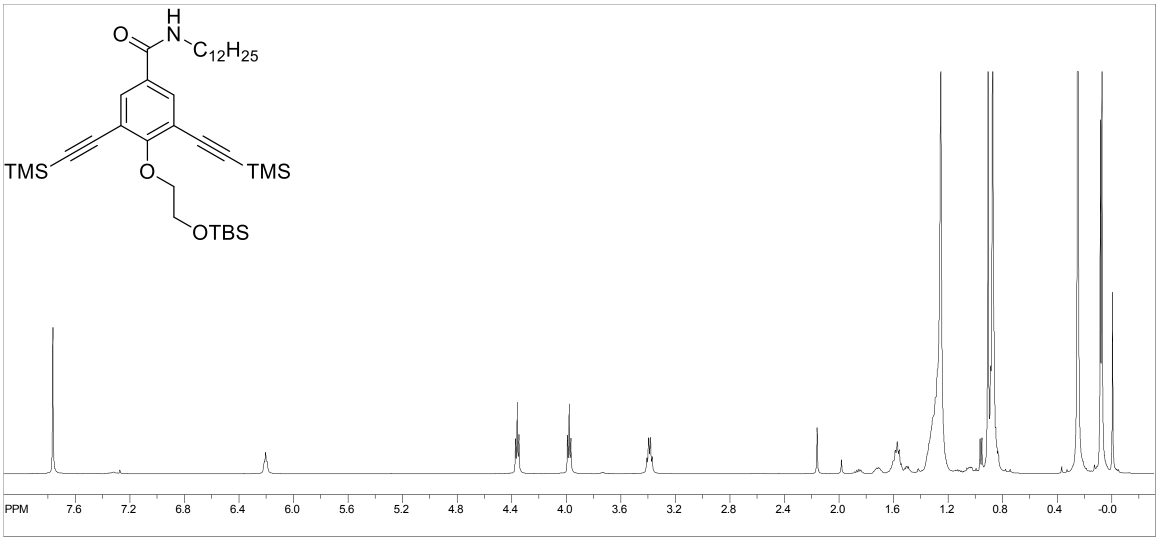

Figure S7. ^{1}H NMR of compound **4** in $CDCl_3$

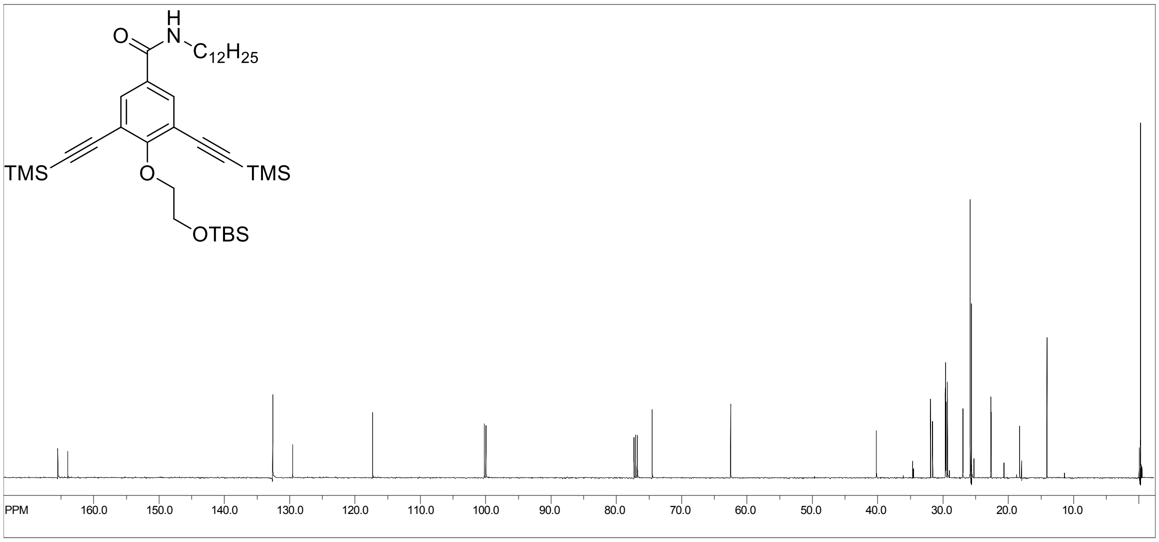

Figure S8. ^{13}C NMR of compound **4** in $CDCl_3$

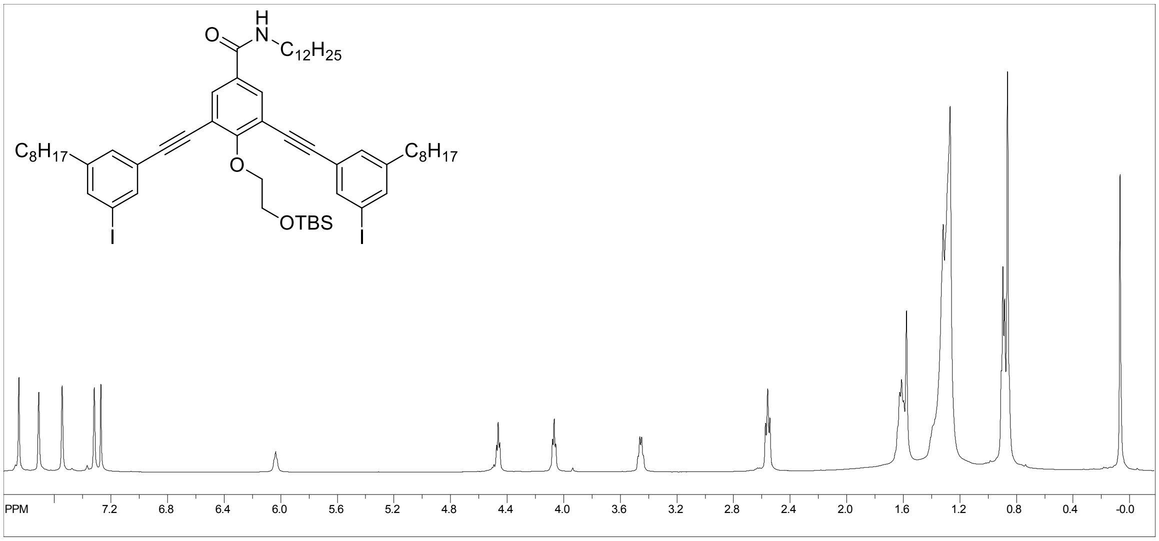

Figure S9. ^{1}H NMR of compound **5** in $CDCl_3$.

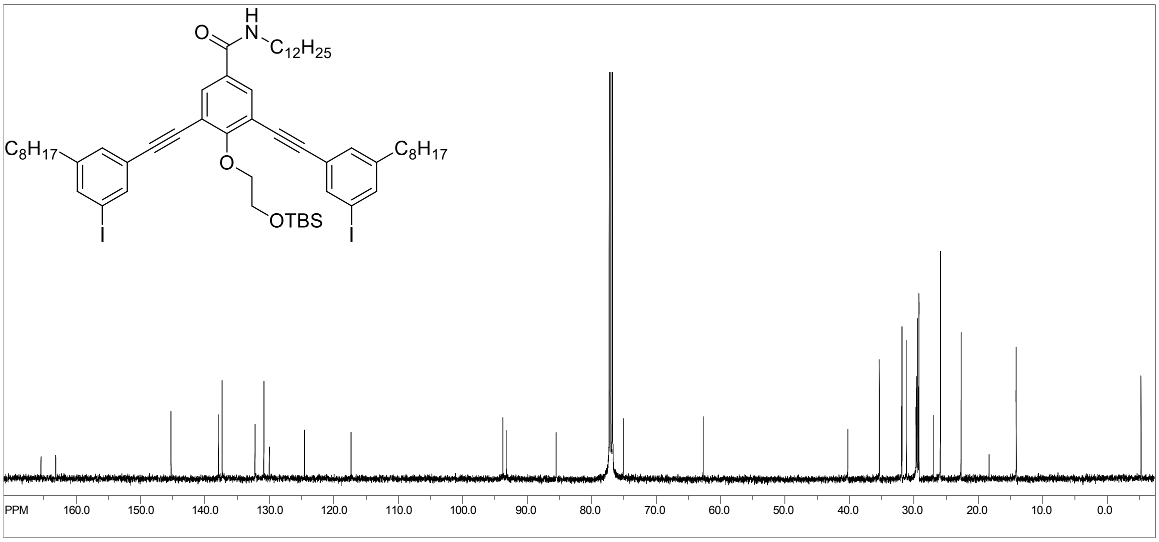

Figure S10. ^{13}C NMR of compound **5** in $CDCl_3$.

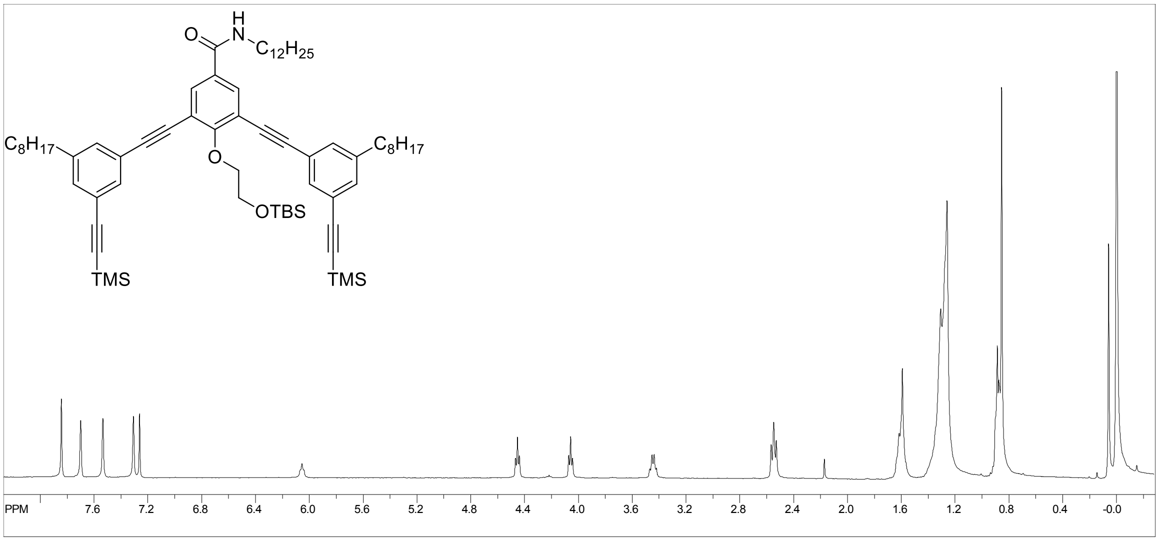

Figure S11. ^{1}H NMR of compound **6** in $CDCl_3$.

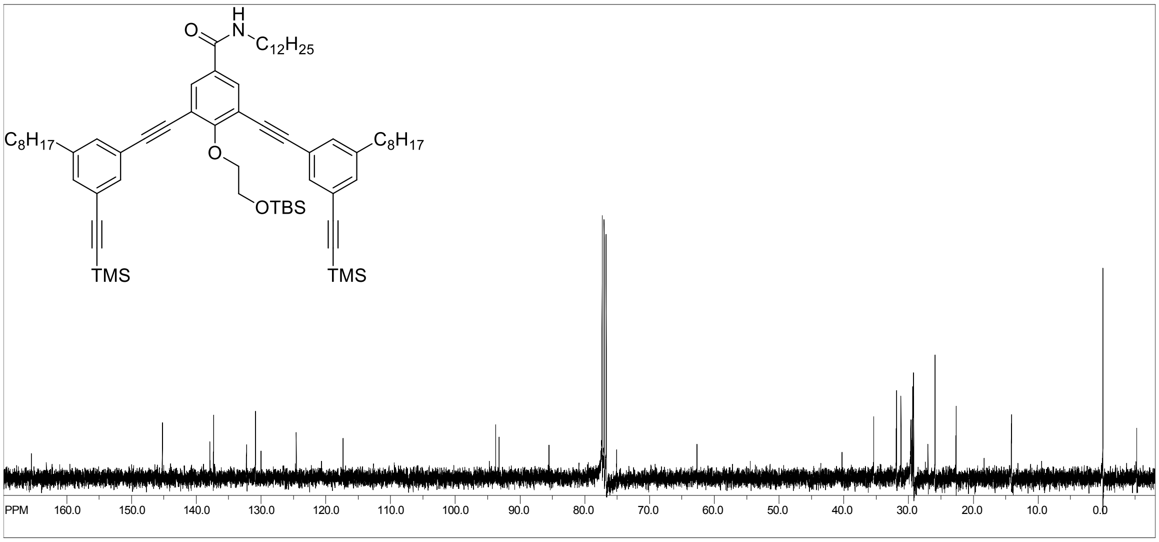

Figure S12. ^{13}C NMR of compound **6** in $CDCl_3$

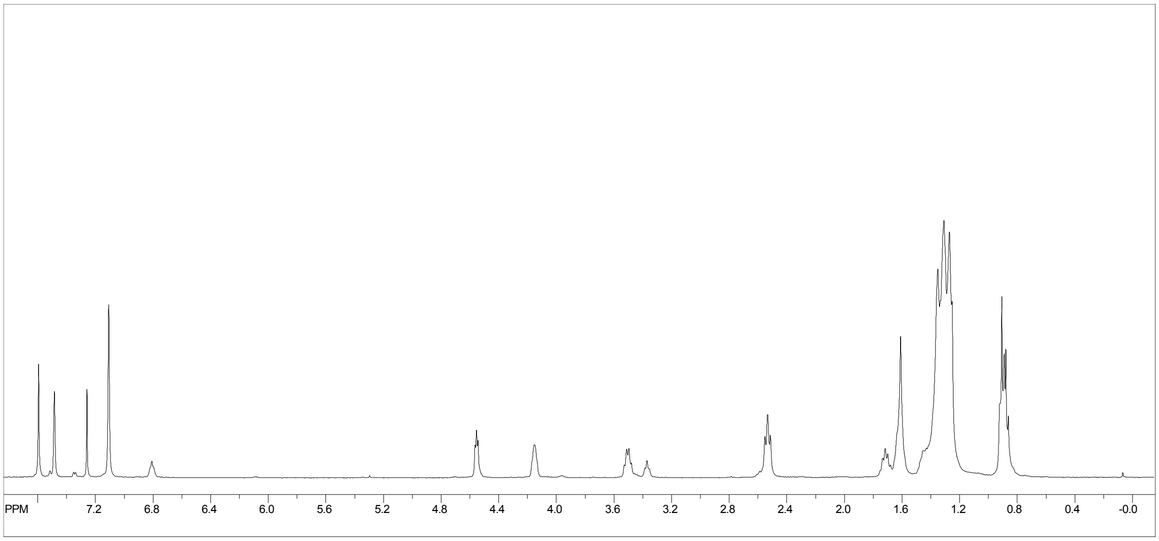

Figure S13. ^{1}H NMR of **PAM2** in $CDCl_3$

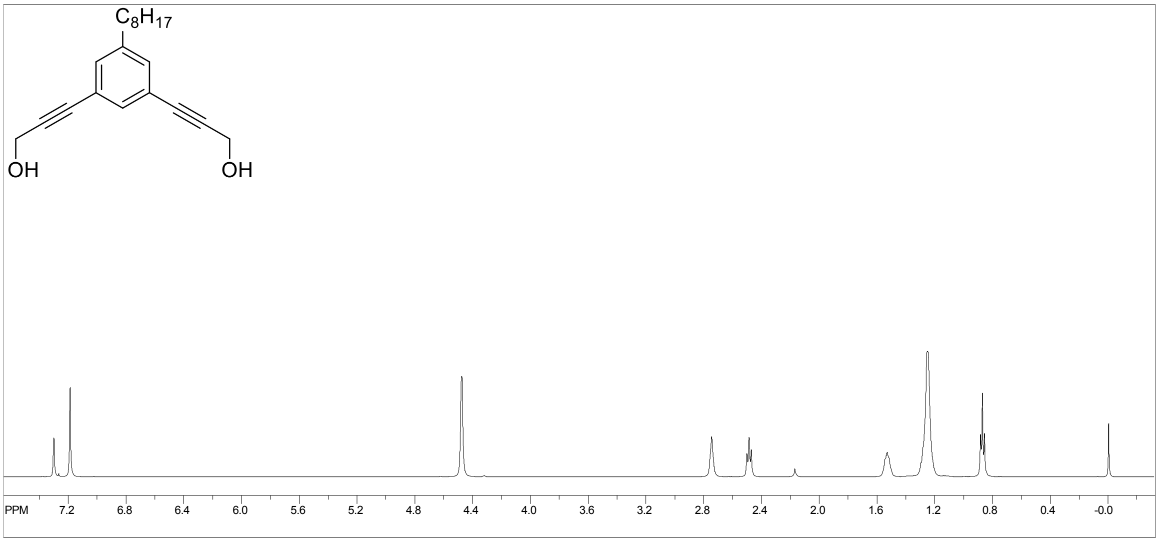

Figure S14. ^{1}H NMR of compound **7** in $CDCl_3$

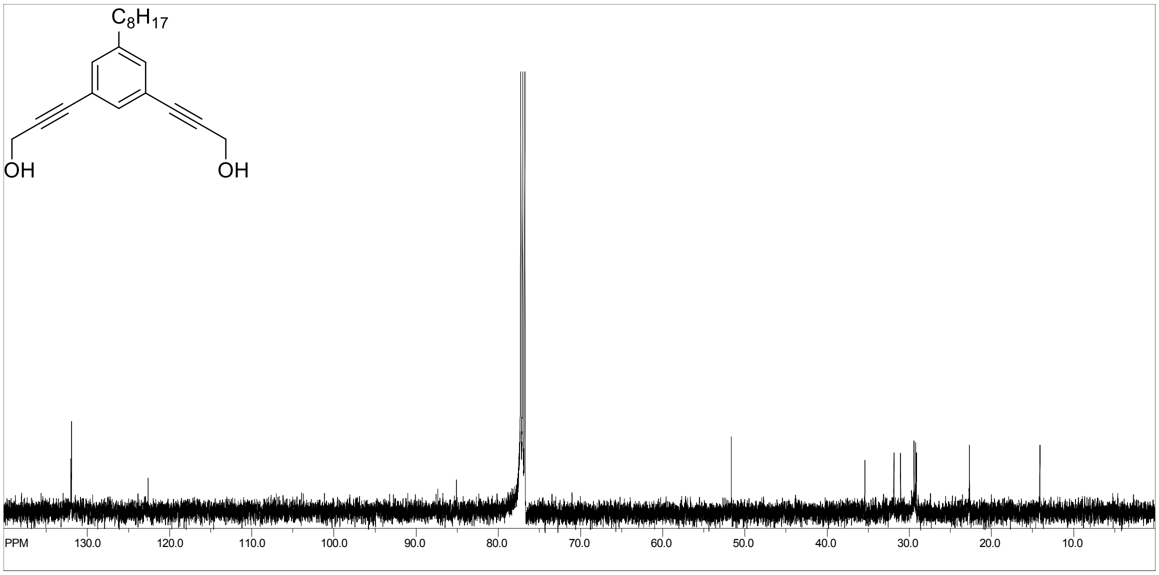

Figure S15. ^{13}C NMR of compound **7** in $CDCl_3$

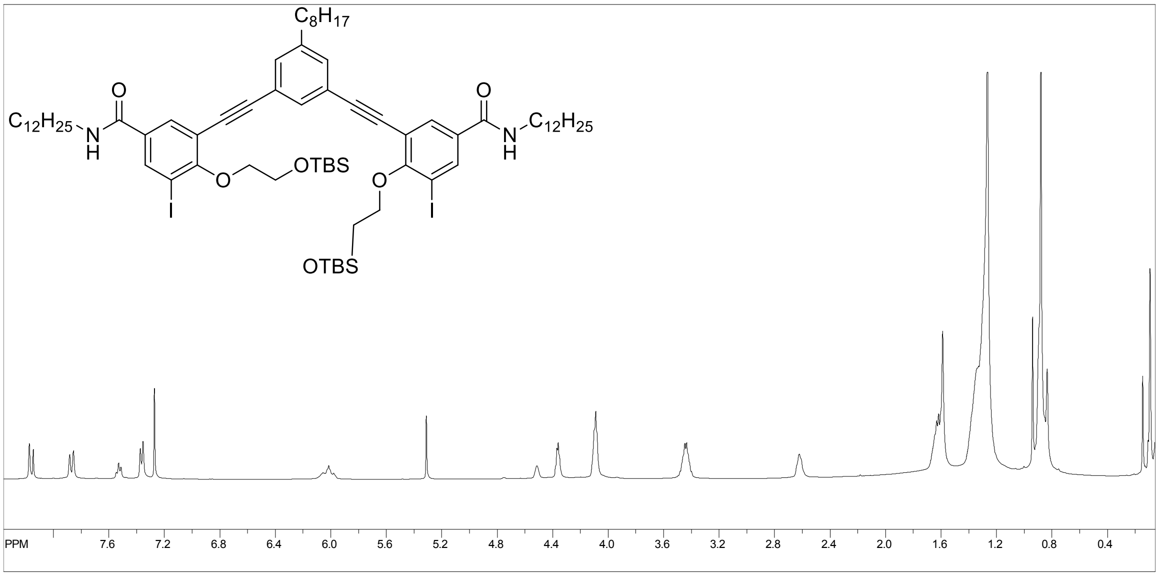

Figure S16. ^{1}H NMR of compound **9** in $CDCl_3$

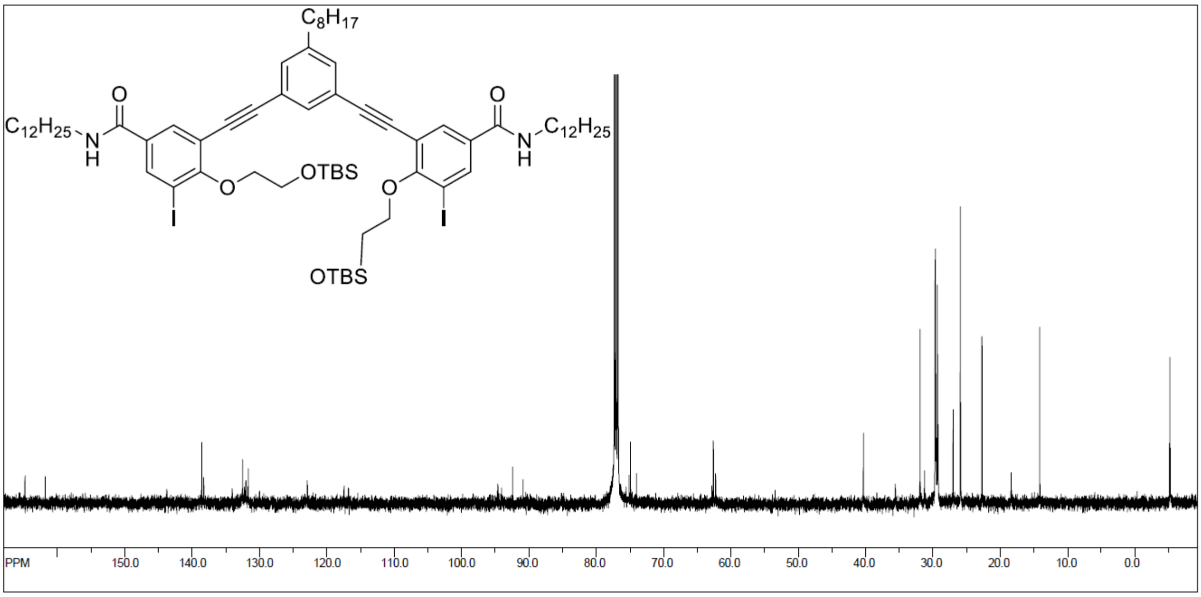

Figure S17. ^{13}C NMR of compound **9** in $CDCl_3$

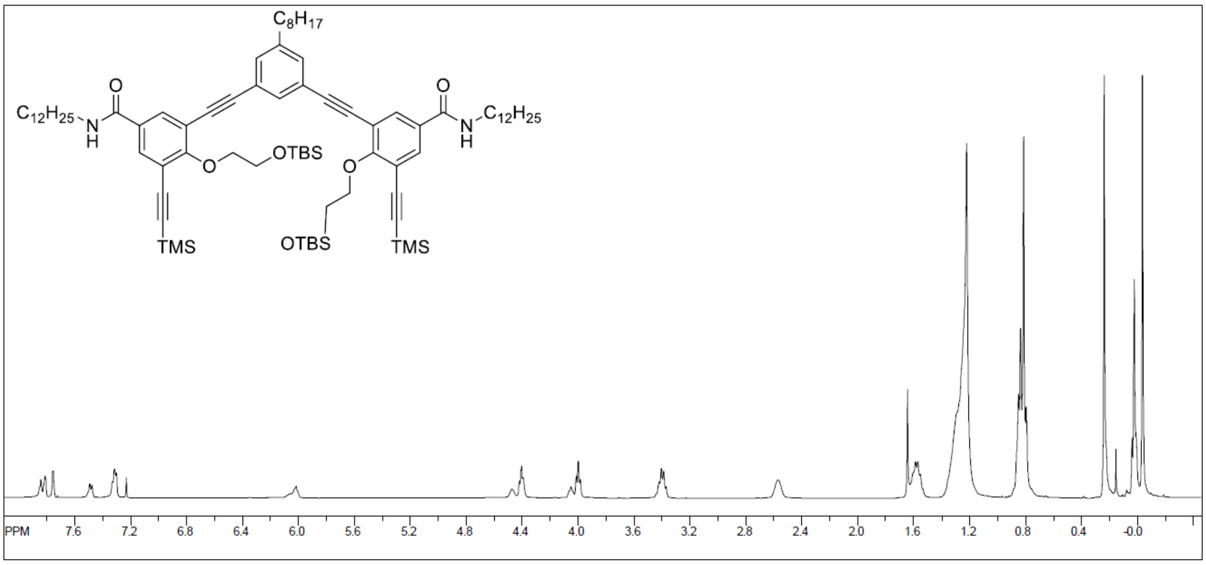

Figure S18. ^{1}H NMR of compound **10** in $CDCl_3$

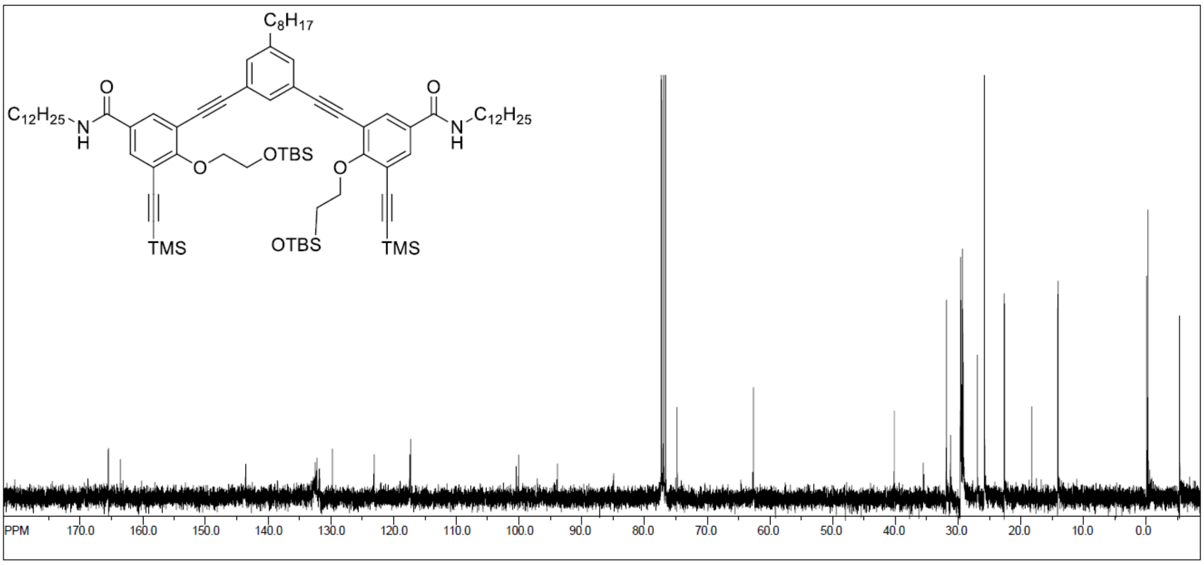

Figure S19. ^{13}C NMR of compound **10** in $CDCl_3$

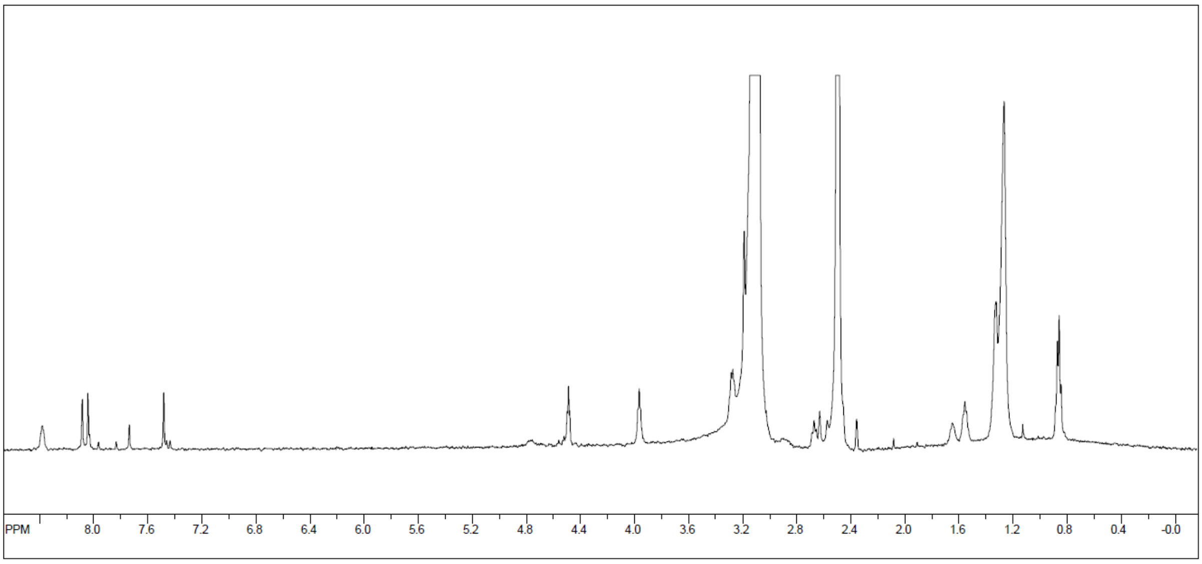

Figure S20. ^{1}H NMR of **PAM3** in DMSO-d_6 at 70 °C

4- Gelation properties

Table S1. Gelation properties of monomers where G = gel; PG= partial gelification, S = solution, I = insoluble, a= previously reported

Solvent	PAM1	PAM2	PAM3
**Toluene**	G	G	I
***o*-DCB**	S	S	I
**Benzene**	G	G	PG
**Cyclohexane**	G	G	PG
**Ethyl Acetate**	G	G	I
**MeOH**	I	I	I
**$CHCl_3$**	S	S	I

5- X-Ray diffraction (PXRD)

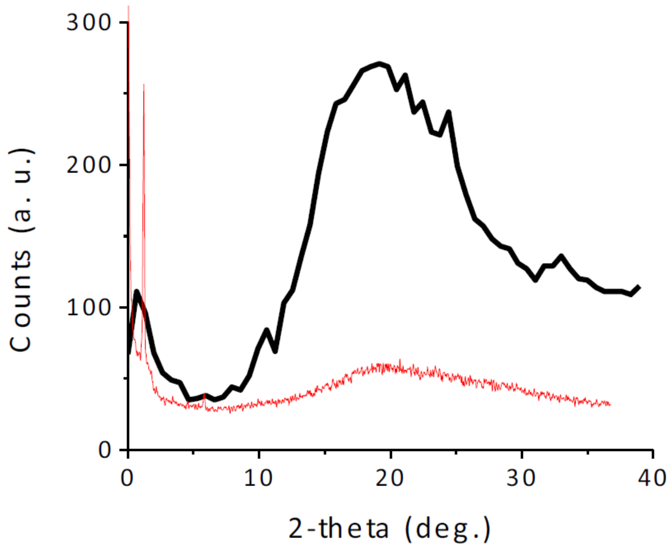

Figure S21. X-ray diffraction (PXRD) spectrum of a 10 mg/mL xerogel of **PAM1** (red line) and **PAM2** (black line) in cyclohexane.[1]

6- Differential scanning calorimetry (DSC)

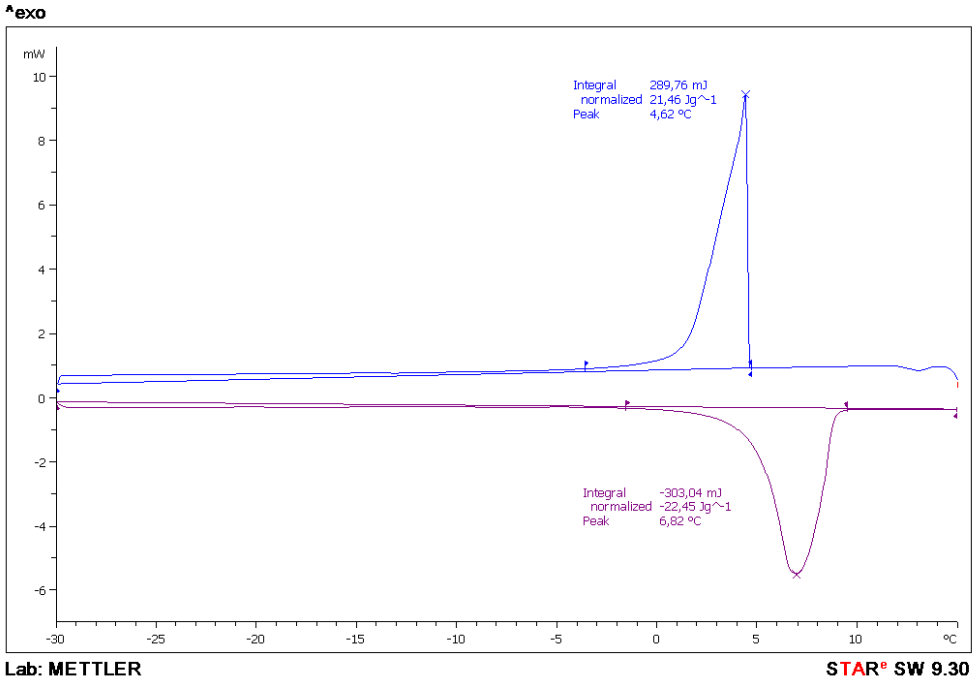

Figure S22. DSC curves of a gel (10 mg/mL) of **PAM1** in cyclohexane, 10°C/min.

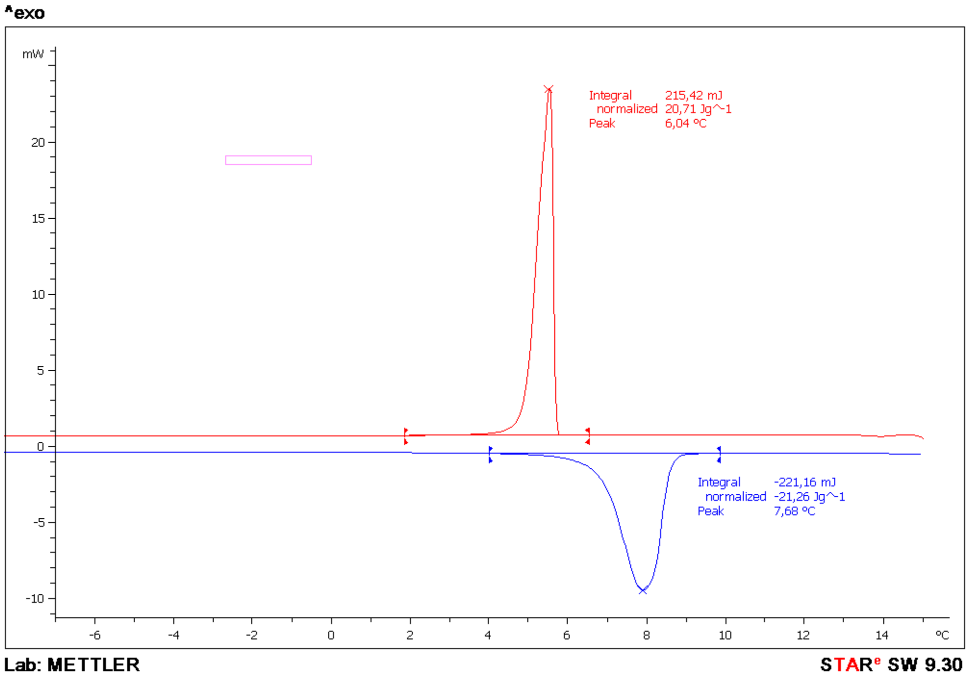

Figure S23. DSC curves of a gel (10 mg/mL) of **PAM2** in cyclohexane, 10°C/min.

7- Transmission electron microscopy (TEM)

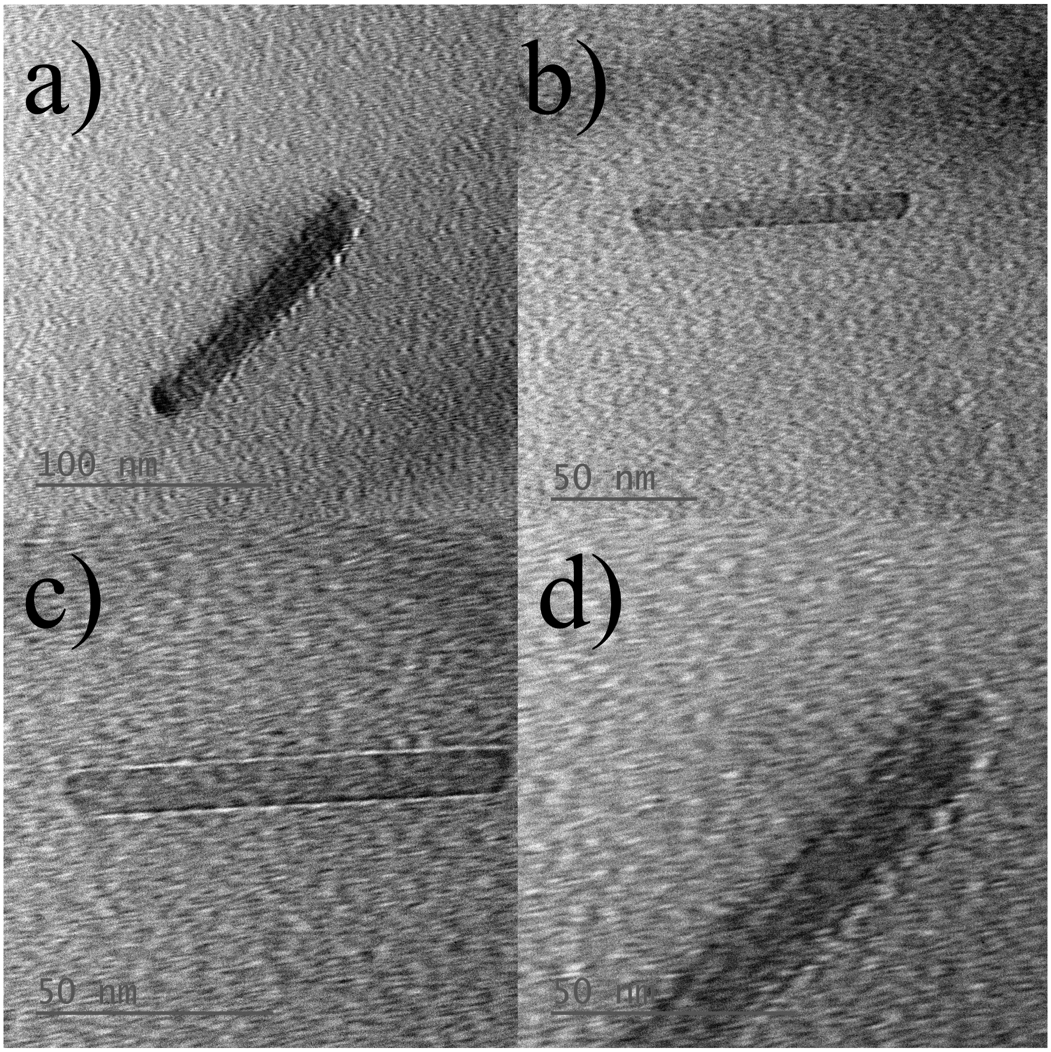

Figure S24**.** TEM imaging of PDA on carbon-coated copper grid. Scale bars are a) 100 nm and b) to d) 50 nm.

8 – References

1 Cantin, K.; Rondeau-Gagné, S.; Néabo, J. R.; Daigle, M.; Morin, J.-F. *Org. Biomol. Chem.* **2011**, *9*, 4440.
